# Hyperglycemia enhances brain susceptibility to lipopolysaccharide-induced neuroinflammation via astrocyte reprogramming

**DOI:** 10.1186/s12974-024-03136-1

**Published:** 2024-05-27

**Authors:** Kyung-Seo Lee, Sung-Hyun Yoon, Inhwa Hwang, Jeong-Hwa Ma, Euimo Yang, Rebekah Hyeyoon Kim, Eosu Kim, Je-Wook Yu

**Affiliations:** 1 Department of Microbiology and Immunology, Institute for Immunology and Immunological Diseases, Yonsei University College of Medicine, Seoul, Korea; 2 Graduate School of Medical Science, Brain Korea 21 Project, Yonsei University College of Medicine, Seoul, Korea; 3 Department of Psychiatry, Institute of Behavioral Science in Medicine, Yonsei University College of Medicine, Seoul, Korea

**Keywords:** Hyperglycemia, Neuroinflammation, Astrocytes, Microglia, Lipopolysaccharide, RNA-sequencing, Cognitive function

## Abstract

Hyperglycemia has been shown to modulate the immune response of peripheral immune cells and organs, but the impact of hyperglycemia on neuroinflammation within the brain remains elusive. In the present study, we provide evidences that streptozotocin (STZ)-induced hyperglycemic condition in mice drives a phenotypic switch of brain astrocytes to a proinflammatory state, and increases brain vulnerability to mild peripheral inflammation. In particular, we found that hyperglycemia led to a significant increase in the astrocyte proliferation as determined by flow cytometric and immunohistochemical analyses of mouse brain. The increased astrocyte proliferation by hyperglycemia was reduced by Glut1 inhibitor BAY-876. Transcriptomic analysis of isolated astrocytes from *Aldh1l1*^*CreERT2*^;*tdTomato* mice revealed that peripheral STZ injection induced astrocyte reprogramming into proliferative, and proinflammatory phenotype. Additionally, STZ-induced hyperglycemic condition significantly enhanced the infiltration of circulating myeloid cells into the brain and the disruption of blood-brain barrier in response to mild lipopolysaccharide (LPS) administration. Systemic hyperglycemia did not alter the intensity and sensitivity of peripheral inflammation in mice to LPS challenge, but increased the inflammatory potential of brain microglia. In line with findings from mouse experiments, a high-glucose environment intensified the LPS-triggered production of proinflammatory molecules in primary astrocyte cultures. Furthermore, hyperglycemic mice exhibited a significant impairment in cognitive function after mild LPS administration compared to normoglycemic mice as determined by novel object recognition and Y-maze tasks. Taken together, these results demonstrate that hyperglycemia directly induces astrocyte reprogramming towards a proliferative and proinflammatory phenotype, which potentiates mild LPS-triggered inflammation within brain parenchymal regions.

**Supplementary Information:**

The online version contains supplementary material available at 10.1186/s12974-024-03136-1.

## Introduction

Hyperglycemia, characterized by elevated blood glucose levels, is a hallmark feature of diabetes mellitus, a chronic metabolic disorder that affects millions of people worldwide. While the detrimental effects of hyperglycemia on various peripheral organs have long been studied [1, 2], its intricate involvement in driving brain inflammation has gained attention in recent years. This growing interest stems from the realization that hyperglycemia can exert profound effects on the central nervous system contributing to the pathogenesis of neuroinflammatory and neurodegenerative diseases [3–5]. Indeed, patients with type I or type II diabetes exhibit a heightened susceptibility to cognitive impairment and are prone to experiencing more severe cerebral lesions than healthy individuals [6–9]. However, the direct and specific impact of hyperglycemia on the brain-resident cells remains largely unknown. In particular, given the emerging role of immune responses in mediating neurodegenerative disorders [10–13], hyperglycemia-induced changes in brain inflammation remain to be determined.

The brain is predominantly dependent on glucose as its primary energy source, and the optimal maintenance of glucose metabolism is critical for proper brain function [14]. Astrocytes, a subtype of brain glial cells, play a pivotal role in controlling the uptake of glucose from the bloodstream and its subsequent distribution to brain cells [15]. Moreover, astrocytes are implicated in many essential brain homeostatic functions such as neurotransmitter synthesis, synapse formation, and blood-brain barrier (BBB) maintenance [16, 17]. Of interest, astrocytes can produce proinflammatory cytokines and interact with immune cells upon the inflammatory stimulation, thereby actively participating in the regulation of neuroinflammation [18, 19]. In this context, we speculated that astrocytes might play a key role in modulating the inflammatory status within the brain under hyperglycemic conditions.

Recent studies have demonstrated that hyperglycemic conditions exacerbate neuroinflammatory responses mainly associated with traumatic brain injury or ischemic stroke [20–22]. Hyperglycemia is prevalent in the patients with ischemic stroke and also enhances the risk of stroke through various pathological effects [23, 24]. Indeed, morphological changes in brain glial cells have been frequently observed in hyperglycemic conditions [25]. However, the precise impact of hyperglycemia on the inflammatory status of the brain remains largely unknown. In this context, we unexpectedly found that streptozotocin-induced hyperglycemia markedly increases the proliferation of astrocytes, affecting blood-brain barrier function and brain inflammatory status. In the present study, we provide evidences that hyperglycemic conditions induce astrocyte reprogramming towards a proinflammatory phenotype, which increases the brain’s sensitivity to peripheral or systemic inflammatory stimuli.

## Materials and methods

### Mice

C57BL/6 mice were purchased from Orient Bio. *Aldh1l1*^*cre/ERT2*^ (JAX#029655) and *Rosa26-tdTomato*^*fl/fl*^ (Ai14, JAX#007914) mice were obtained from The Jackson Laboratory and bred at Yonsei University College of Medicine. *Aldh1l1*^*cre/ERT2*^ mice and *tdTomato*^fl/fl^ mice were crossed to generate astrocyte-specific tdTomato-expressing mice. *Tmem119-EGFP* mice were provided by Dr. Ki Woo Kim (Yonsei University). All mice were C57BL/6 background and maintained in the specific pathogen-free facility with temperature- and humidity-controlled conditions on a 12 h–12 h light-dark cycle. Age-matched (8–12 weeks old) male mice were used for the experiments. All animal protocols were approved by the Institutional Ethical Committee, Yonsei University College of Medicine (2020 − 0317). All experiments were performed in accordance with the approved guidelines of the Institutional Ethical Committee.

### Mice treatment

Hyperglycemic state in mice was induced by streptozotocin (STZ) administration according to a previous study [26]. Briefly, 8-week-old male mice were fasted for 5 h and then given a single intraperitoneal injection of STZ (150 mg/kg, Sigma-Aldrich, S0130) dissolved in sodium citrate buffer (0.1 M, pH 4.5). The control group received only sodium citrate buffer (vehicle). Five days after injection, blood samples were collected from the mice’s tail vein, and fasting blood glucose levels were measured using an Accu-Chek Performa glucometer (Roche). Mice with blood glucose levels exceeding 250 mg/dl were considered hyperglycemic and selected for the study. To induce peripheral inflammation, mice were injected intraperitoneally with lipopolysaccharide (LPS) (0.5 mg/kg, *E. coli* O111:B4, Sigma-Aldrich, L3012) or an equivalent volume of PBS for the control group. Mice were sacrificed either 3–6 h post PBS or LPS injection for subsequent analysis. For the NOR and Y-maze tests, mice were administered with LPS (0.25 mg/kg) and rested for 24 h before behavioral assessments. Tamoxifen (Sigma-Aldrich) in a corn oil solution was intraperitoneally injected (75 mg/kg) for 5 consecutive days into *Aldh1l1*^*cre/ERT2*^; *tdTomato*^*fl/fl*^ mice. One week after the final tamoxifen injection, appropriate treatments were administered to the mice.

### Tissue preparation for flow cytometry

Mice were euthanized and a transcardial perfusion with PBS was performed to remove circulating blood. Subsequently, the brain tissue was carefully isolated with the olfactory regions, brain stem, and meninges being excised. Then brain was then homogenized in RPMI-1640 containing DNase I and Collagenase IV, followed by an incubation at 37 °C. The resulting homogenates were subjected to Percoll gradient centrifugation to eliminate myelin debris. Blood samples from mice were collected in EDTA (5 mM) using capillary tubes. Red blood cells (RBCs) were lysed using ACK lysing buffer, and the cells were obtained through centrifugation. For bone marrow cell preparation, mouse femur bone was flushed with PBS, followed by lysis of RBCs. The cells are resuspended in an appropriate staining medium for flow cytometric analysis, and cell counts were quantified using hemocytometer before analysis.

### Flow cytometry

Fluorochrome-conjugated monoclonal antibodies targeting CD45 (Biolegend), CD11b (Invitrogen), ACSA2 (Miltenyi), Ly6C (Invitrogen), and Ly6G (Invitrogen) were used to stain cells for flow cytometry analysis. DAPI counterstaining was employed to exclude dead cells. The analyses were conducted using FACSVerse (BD Biosciences) and FlowJo software (TreeStar). Total immune cells, encompassing both myeloid and lymphoid cells, were identified as CD45-positive cells. CD45^+^ cells were further categorized into brain-resident microglia and brain-infiltrating myeloid cells such as neutrophils and monocytes based on CD11b expression. For the isolation of microglia and astrocytes from the mouse brain, cells were sorted from Tmem119-EGFP (microglia) and Aldh1l1-cre/ERT; tdTomato^fl/fl^ (astrocytes) using either FACS Aria II or FACS Aria III cell sorter (BD Biosciences).

### RNA-Seq analysis

For the transcriptomic analysis of astrocytes, mouse brain samples underwent enzymatic cell dissociation using an Adult Brain Dissociation Kit (Miltenyi Biotec), according to the manufacturer’s instructions. The resulting dissociated samples were collected and filtered through a 70-µm cell strainer. Debris Removal Solution was applied to eliminate myelin debris, and Red Blood Cell Removal Solution was used to clear red blood cells. Cells from three-independent brains were pooled for each sample.

RNA sequencing was carried out by Macrogen using the manufacturer’s reagents and protocols. Illumina TruSeq RNA library Preparation v2 kit was employed for the preparation of sample libraries, and SMART-Seq v4 Ultra Low Input RNA for Sequencing was used for RNA-seq. The resulting count files (fastq) were downloaded and aligned to the *M.musculus* mm10 genome using HISAT2 program, with transcript assembly performed using the StringTie program. Outliers were excluded from each sample’s potential outlier genes for the analysis, and genes with zero read counts in any sample were excluded. DEG (Differentially Expressed Genes) analysis was performed using DESeq2 program, with most comparisons and analyses conducted using an FDR-adjusted *p*-value < 0.05, unless specified otherwise. Heatmaps and PCA plots were generated using the Macrogen DEG viewer program or R project, bar plots were generated using R project, and Gene Set Enrichment Analysis (GSEA) on Gene Ontology (GO) enrichment was performed using the GSEA tool (https://www.gsea-msigdb.org/gsea/index.jsp).

### Evans blue leakage assay

Evans blue (Sigma) was dissolved in PBS and intravenously administered to mice via tail vein (0.2 mg/kg). After 1 h, mice were deeply anesthetized and transcardially perfused with PBS. Subsequently, brain tissues were collected and homogenized using trichloroacetic acid (Sigma, T6399). Following centrifugation, the absorbance of the supernatants was measured at 620 nm excitation and 680 nm emission using the Varioskan Flash 3001 microplate fluorometer (Thermo Fisher). The BBB permeability was evaluated by quantifying the amount of Evans blue leakage.

### Immunohistochemistry

Mice were euthanized, and the brains were perfused with PBS and subsequently fixed with 4% paraformaldehyde. After overnight fixation, brain tissue samples were immersed in 30% sucrose at 4 °C for 2 days until the samples sunk to the bottom. Tissues were then embedded in OCT compound fully frozen on dry ice. Coronal Sect. (30 μm thickness) of the embedded brain tissues were obtained using Leica CM1860 cryotstat. The frozen sections were mounted on glass slides, treated with permeabilization buffer (0.3% Triton X-100 in PBS) for 30 min at room temperature (RT), and then exposed to a block solution (4% BSA in PBS) for 2 h. Subsequently, the sections were incubated with primary antibodies targeting GFAP (Invitrogen), Iba1 (Wako), and BrdU (Abcam) overnight at 4 °C, followed by treatment with Cy3-, FITC-, or Alexa 488-labeled secondary antibodies. For BrdU detection, samples were incubated with 2 N HCl (30 min, RT) prior to permeabilization. DAPI counterstaining was used to visualize the nuclei. Consistent regions of the hippocampus were obtained from each sample within the mouse group. Z-stack images, with a 20 μm span at 1 μm intervals, were generated through orthogonal projection. All images were acquired and processed with confocal microscopy (LSM980, Carl Zeiss) and Zen blue software (v.3.6.095).

### Quantification of immunohistochemical images

Image post-processing and analyses of individual cell numbers and fluorescence intensity were performed using Image J software. Image post-processing parameters were kept consistent across all control and experimental groups for comparison. Brightness and contrast were adjusted to allow for visualization and analyses. Quantification of astrocytes and microglia was determined by GFAP^+^ and Iba1^+^ cells, respectively, normalized by dividing the values by DAPI^+^ cells per sample of brain hippocampal image. The threshold was optimized to a constant value for measurement of GFAP and Iba1 mean fluorescence intensity. Morphological analyses of microglia were performed using Imaris software (v.9.7). For 3D reconstruction of microglia, the Z-stack images (20 μm depth, 1 μm intervals, x40 magnification) were acquired and imported in Imaris for visualization.

### Cell culture

Mouse bone marrow cells were isolated from femurs and tibias of mice and cultured in L929-conditioned Dulbecco’s modified Eagle’s medium (DMEM) for 5–7 days for differentiation into BMDMs [27]. To isolate resident peritoneal macrophages, we collected mouse peritoneal lavage by rinsing with PBS containing 2% FBS. The peritoneal cells were then harvested by centrifugation, resuspended in RPMI1640 media, and allowed to adhere to a cell culture dish. Following overnight incubation, the adherent cells were washed three times with warm PBS and employed as peritoneal macrophages. Mouse brain mixed glial cells were prepared from the whole brains of mouse pups on postnatal days 0–2 as described previously [28] and cultured in normoglycemic (5.5 mM glucose) or hyperglycemic DMEM (25 mM glucose) supplemented with 10% FBS and antibiotics for 7 days. Astrocytes were then isolated from the mixed glial cells by removal of microglia using mild trypsinization (150 rpm, 6 h).

### Enzyme-linked immunosorbent assay (ELISA)

Serum from mice was obtained through centrifugation of whole blood samples and then appropriately diluted for ELISA assay. In the case of cultured cells, the media was collected and subjected to centrifugation to remove debris and cellular components. The concentrations of IL-1β, TNF-α, and IL-6 were determined using IL-1β, TNF-α, IL-6 ELISA DuoSets kit (R&D systems), respectively, in accordance with manufacturer’s instructions.

### Quantification of mRNA

For evaluation of the mRNA transcription level, total RNA was isolated from sorted cells, brain tissue, and spleen tissue using TRIzol reagent (Invitrogen), and then was reversely transcribed using Reverse Transcriptase primer mix (Takara). Quantitative real-time PCR was performed using SYBR Premix Ex Taq (Takara) for comparative analysis of gene expression, in which Rn18s was amplified as the control gene. Primers were as follows: 5’-GCC CAT CCT CTG TGA CTC AT-3’ and 5’-AGG CCA CAG GTA TTT TGT CG-3’ (mouse *il-1*𝝱); 5’-AGG TCA TTG GTG GAG AGG TG-3’ and 5’-CCT GCT TGA GTA TGT CGC AC-3’ (mouse *cox-2*); 5’-CCA AGT TCC ACC TGC TCA AC-3’ and 5’-GCC GAG CTG ACA CCT TAA TG-3’ (mouse *pla2g3*); 5’-CGG ACT CCG CAA AGT CTA AG-3’ and 5’-CGT CAG CCG ATT TGC TAT CT-3’ (mouse *tnf*-𝝰); 5’-GTT TCT GGG GAG AGG GTG AG-3’ and 5’-TGT TCT ACT CTC CTC GGT GC-3’ (mouse *cxcl2*); 5’-TTG CTT TGG ACT CAG CAT TG -3’ and 5’-GGG AGG TGT GAC CAG GTA GA-3’ (mouse *aqp4*); 5’-GGG TCT ACA CCA GCA AGA GC-3’ and 5’-ACC GAG ACA TCA GGG CAT AC-3’ (mouse *axl*); 5’ -TCG ACC GAA TCC TGA GTA GC-3’ and 3’ -CGT CCT TTG TCA CTT GCA CA -3’ (mouse *pfkp*); 5’ -CTG GAA TGA ATG TGG CTC GG-3’ and 5’ -TAA GCG TTG TCC AGG GTG AT-3’ (mouse *pkm2*); 5’ -TGC ATC CCA TTT CCA CCA TG-3’ and 5’ -GTG TCT GCG CTC TTC TTC AG-3’ (mouse *ldha*); 5’ -ACC ACG GAC TAC AAC CAG TT-3’ and 5’ -CCT TGA GGC CCA GAG ACT TG-3’ (mouse *lcn2*); 5’-CGC GGT TCT ATT TTG TTG GT-3’ and 5’-AGT CGG CAT CGT TTA TGG TC-3’ (mouse *Rn18s*).

### Behavioral test

Behavioral tests were conducted as described in previous study with slight modifications [29]. For Novel Object Recognition (NOR) test, mice were habituated to the open field arena one day before object exposure. During this habituation, mice explored the empty arena for 10 min, and their movements were recorded using a video system. In the training phase, two identical objects were placed in the arena, and mice were allowed to freely explore for 10 min. During the subsequent testing phase (one hour after the training session), one object was replaced with a novel one, and mice were allowed to explore for 5 min. The time spent exploring each familiar and novel object was tracked using the video recording system. The discrimination index was calculated as follows: [time with novel object – time with familiar object] / [time with novel object + time with familiar object]. NOR tests were conducted both before (baseline) and after LPS injection. Mice that did not explore both objects during the training phase were excluded from the analysis.

In the Y-maze task, the three arms of the maze were clearly designated before usage. Mice were placed at the center of the maze, and their spontaneous behavior was recorded for a period of 10 min. The maze was cleaned between each mouse’s task run to eliminate any odor cues. An arm entry was defined as when all four paws of a mouse crossed the threshold of the center zone into the arm, with the snout oriented toward the end of the arm. Spontaneous alternation (%) was calculated as follows: [number of spontaneous alternations] / [total number of arm entries – 2] × 100, where a spontaneous alternation occurred when the mouse entered a different arm in each of three consecutive arm entries. Mice with total arm entries fewer than 10 were excluded from the calculation.

### Statistical analysis

All values were presented as the mean and standard error of the mean (SEM) of individual samples. Data were analyzed using unpaired Student’s *t* tests (two-tailed), one-way or two-way analysis of variance (ANOVA) with Bonferroni post hoc test for comparison of two or more groups. The level of statistical significance was set at *p* ≤ 0.05. Analyses were performed using GraphPad Prism 9.

## Results

### Streptozotocin-induced hyperglycemia increases a population of astrocytes in the brain

**Fig. 1:**
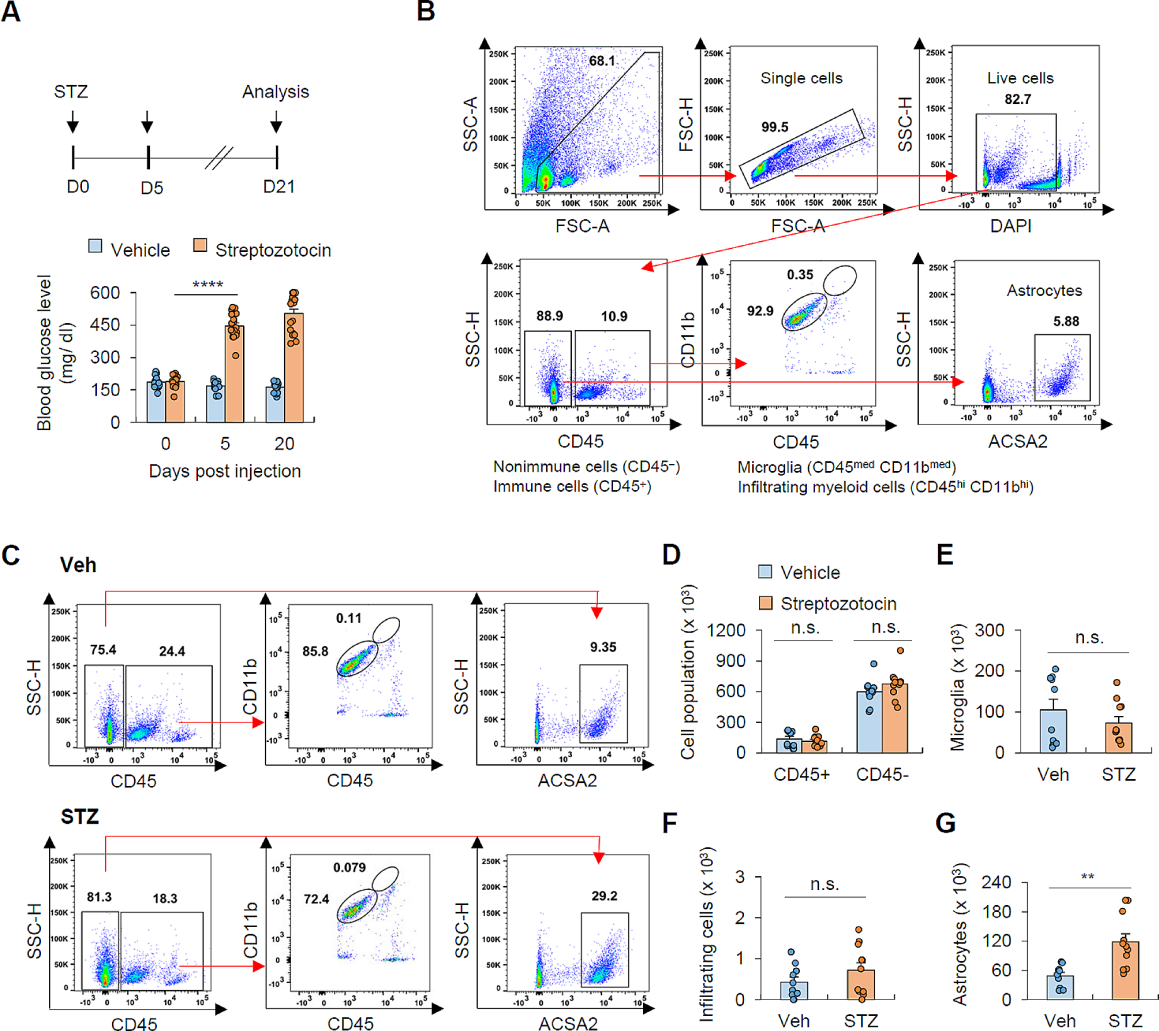
Hyperglycemic condition induces a significant increase in the number of astrocytes (**A**) Experimental scheme for hyperglycemia induction (top) and blood glucose level of mice (bottom) subjected to streptozotocin (STZ) administration (14 mice, vehicle; 18 mice, STZ group). (**B**) Representative gating strategy to identify singlets, live cells (DAPI), microglia (CD45^med^ CD11b ^med^), infiltrated myeloid cells (CD45^hi^ CD11b^hi^), and astrocytes (CD45^−^ ACSA2^+^). (**C**) Flow cytometric analysis of brain tissues from vehicle- or STZ-injected mice. (**D**) Quantification of total immune (CD45^+^) and non-immune (CD45^−^) cells from vehicle- or STZ-treated mice. (10 mice, vehicle; 11 mice, STZ group) (**E**-**G**) Quantification of microglia (**E**), brain-infiltrating myeloid cells (**F**) and astrocytes (**G**) from vehicle- or STZ-treated mice. (10 mice, vehicle; 11 mice, STZ group) Data are presented as means ± SEM. Asterisks indicate significant differences in one-way ANOVA with Bonferroni post hoc test (**A**) and Student’s two-tailed, unpaired *t* test (D-G). **P < 0.01, ****P < 0.0001, n.s. not significant

To explore the impact of hyperglycemia on brain parenchymal cells, we first induced hyperglycemia in mice through a single administration of streptozotocin (STZ). This resulted in the elevated blood glucose levels five days post-injection, which persisted for at least three weeks (Fig. 1A). Additionally, we observed a reduction in body weight in mice with STZ-induced hyperglycemia (Additional file 1: Fig. S1A). Subsequently, we analyzed hyperglycemia-induced changes in brain cells using flow cytometry to assess brain cell populations (Fig. 1B). STZ-induced hyperglycemia did not cause significant alterations in the total number of immune cells (CD45^+^) and non-immune cells (CD45^−^) in the brain (Fig. 1C, D). Likewise, the populations of brain-resident microglia (CD45^med^CD11b^med^) and brain-infiltrating myeloid cells (CD45^hi^CD11b^hi^) remained unaffected in response to hyperglycemic conditions (Fig. 1E, F). However, we observed a notable and significant increase in the number of astrocytes (CD45^−^ACSA2^+^) in the hyperglycemic group (Fig. 1G).

**Fig. 2:**
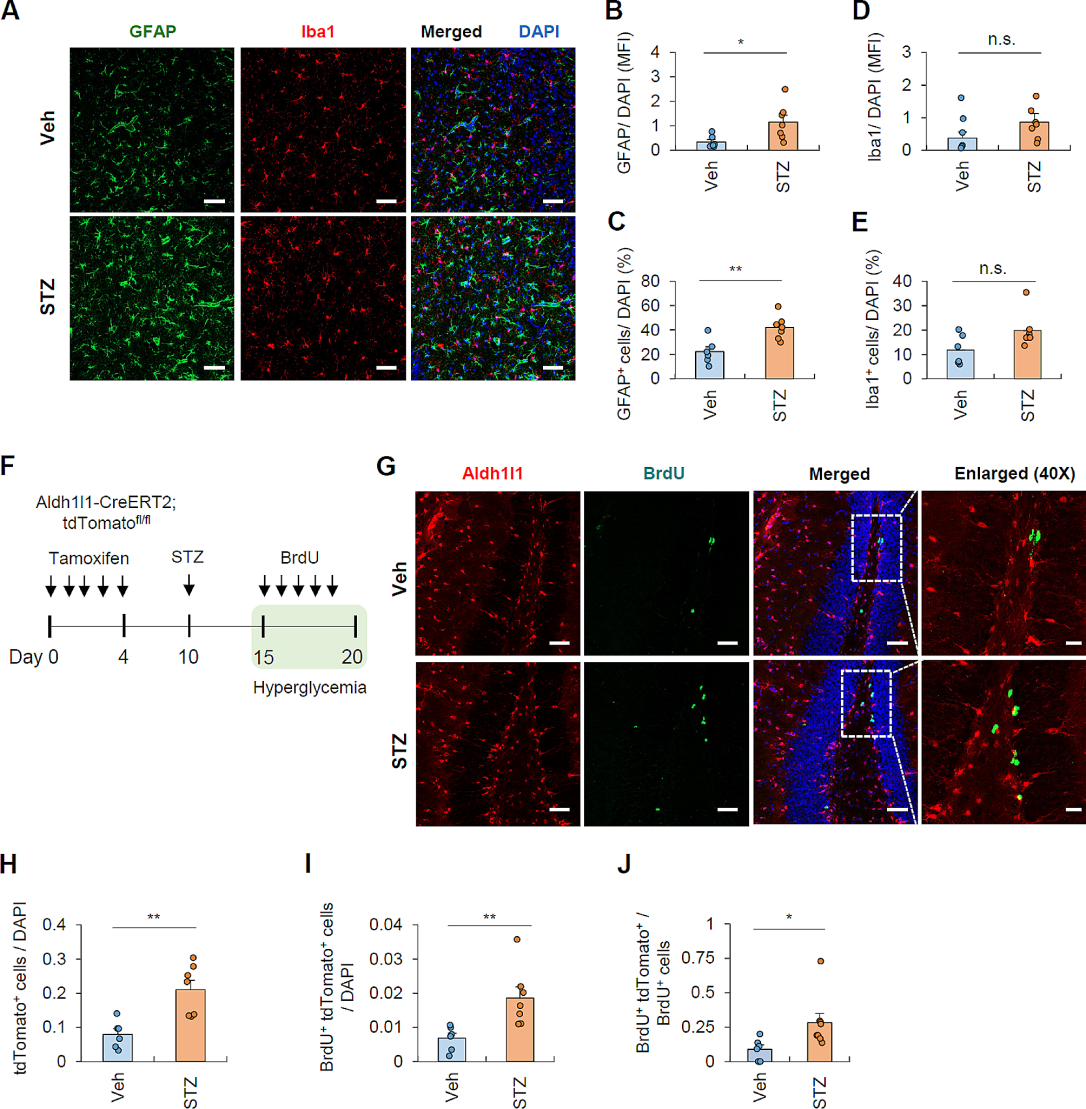
Hyperglycemic condition induces astrogliosis and the proliferation of astrocytes (**A**) Representative immunohistochemical images of hippocampal regions of vehicle- or STZ- injected mice. The coronal sections of brain were stained with anti-GFAP (green) and anti-Iba1 (red). DAPI represents nuclear signal (blue). Scale bars = 50 μm. (**B**-**E**) Quantification of the mean fluorescence intensity (MFI) of GFAP and Iba1 normalized with DAPI (**B** and **D**), and the number of astrocytes (GFAP^+^ cells, C) and microglia (Iba1^+^ cells, E) normalized with DAPI^+^ cells. (6 mice, vehicle; 7 mice, STZ group). (**F**) Experimental scheme for BrdU-based cell proliferation assay. *TdTomato*^*fl/fl*^;*Aldh1l1*^*CreERT2*^ mice were subjected to tamoxifen administration for five days and injected with STZ (d10) to induce hyperglycemia. After another five days, mice were injected with BrdU (d15, 50 mg/kg) for five consecutive days. (**G**) Immunohistochemical analysis of BrdU cell proliferation assay in the hippocampal region of vehicle- and STZ-injected mice. (6 mice, vehicle; 7 mice, STZ group) Cells were stained with anti-BrdU (green) and astrocytes were labeled by tdTomato (red). DAPI represents nuclear signal (blue). The boxed areas indicate the colocalization of BrdU and astrocytes. (H-I) Quantification of astrocytes (tdTomato^+^ cells, H) and proliferating astrocytes (BrdU^+^ tdTomato^+^ cells, I) normalized by DAPI count. (**J**) Quantification of relative BrdU-expressing astrocytes per total BrdU^+^ cells. Scale bars = 50 μm (20X) and 20 μm (enlarged, 40X). Asterisks indicate significant differences in Student’s two-tailed, unpaired *t* test. *P < 0.05, **P < 0.01, n.s. not significant

Next, we examined brain glial cells by conducting immunohistochemical analysis on the hippocampal region of mouse brains. We used antibodies targeting GFAP and Iba1, which specifically label astrocytes and microglia, respectively (Fig. 2A; Additional file 1: Fig. S1B). In line with the findings from flow cytometric analysis, immunohistochemical staining of coronal sections revealed a marked increase in both fluorescence intensity and cell number of GFAP-positive astrocytes in hyperglycemic brains compared to the control group (Fig. 2B, C). Conversely, the fluorescence intensity of Iba1 and cell number of microglia remained unaffected by STZ-induced hyperglycemic conditions (Fig. 2D, E). These results demonstrate that peripheral hyperglycemia specifically leads to an increase in the number of brain astrocytes in the brain.

We then examined whether hyperglycemia could induce the proliferation of astrocytes, as determined by the incorporation of 5’-bromo-2’-deoxyuridine (BrdU). Using *Aldh1l1*^*CreERT2*^; *tdTomato*^*fl/fl*^ mice, we labeled astrocytes with tdTomato by tamoxifen injection. Then, five days after STZ injection, BrdU was administered daily for an additional five days (Fig. 2F). BrdU can only be incorporated into dividing cells. Intriguingly, STZ injection led to a significant increase in BrdU-containing tdTomato-positive astrocytes (Fig. 2G; Additional file 1: Fig. S2), along with the increased number of astrocytes in the hippocampal region of the brain (Fig. 2H). STZ-induced hyperglycemia significantly elevated both the intensity and the proportion of BrdU-expressing astrocytes (Fig. 2I, J). These findings provide additional confirmation that peripheral hyperglycemic conditions induce the proliferation of astrocytes in the hippocampal region of the brain.

### Streptozotocin-induced hyperglycemia induces a phenotype change of astrocytes

**Fig. 3:**
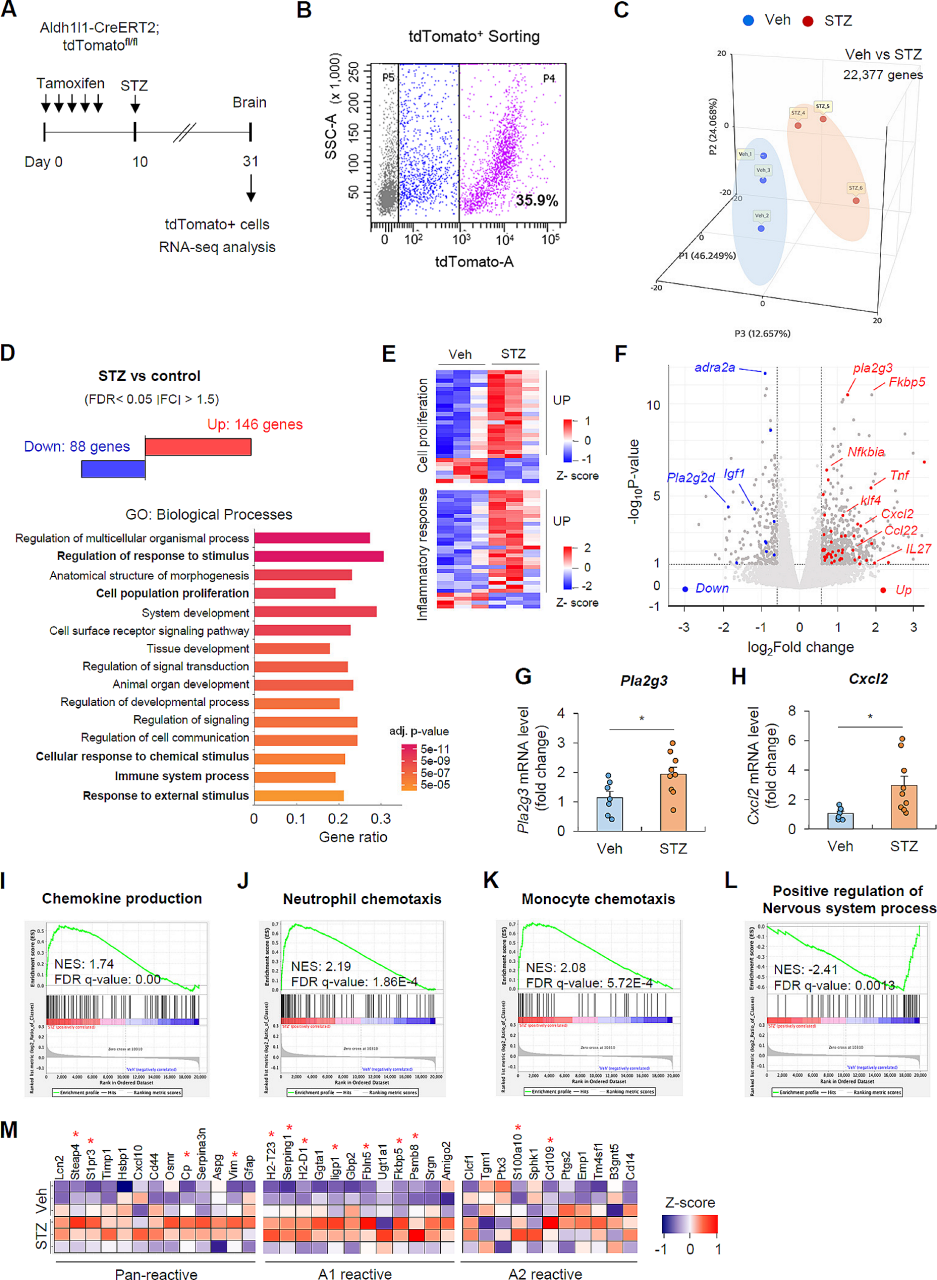
Hyperglycemic condition induces a phenotypic change of astrocytes (**A**) Experimental scheme for RNA-seq analysis of astrocytes from control and STZ-treated mice (*n* = 3 per experimental group, each sample was pooled from 3-independent mice). (**B**) Representative image of tdTomato^+^ astrocytes from brain single cell suspensions of *tdTomato*^*flfl*^;*Aldh1l1-CreERT* mice. (**C**) Distribution of transcriptional differences of vehicle- and STZ-treated astrocytes using 3-dimensional principal component analysis (PCA). (**D**) RNA-seq analysis of differentially expressed genes (DEGs) (FDR adjusted *p*-value < 0.05, |fold change| > 1.5) in STZ-induced hyperglycemic astrocytes. Top 15 Gene Ontology (GO) terms associated with biological processes changed in astrocytes upon hyperglycemic condition in the order of increasing FDR adjusted *p*-value; gene ratio = intersection size/ query size. (**E**) Heatmaps of selected GO terms, positive regulation of cell proliferation (GO:0008284) and inflammatory response (GO:0006954) with colors indicating Z scores. (**F**) Volcano plot depicting the upregulated and downregulated DEGs in astrocytes of STZ-treated mice. Genes associated with inflammatory responses are colored in red and blue. (G-H) Quantification of mRNA levels of *Pla2g3* (**G**) and *Cxcl2* (**H**) in the whole brain extracts of vehicle- and STZ- treated mice. (7 mice, vehicle; 9 mice, STZ group) (**I**-**L**) GSEA (gene set enrichment analysis) analysis of hyperglycemic astrocytes in relation to the control group. (**M**) Heatmap of pan-reactive, A1 reactive, and A2 reactive genes in vehicle- and STZ- treated astrocytes based on RNA-seq analysis. Data are presented as means ± SEM. (**G**, **H**, **M**) Asterisks indicate significant differences. *P < 0.05

To gain molecular insights into the hyperglycemia-induced alterations in brain astrocytes, we conducted a RNA-sequencing analysis on isolated brain astrocytes derived from *Aldh1l1*^*CreERT2*^; *tdTomato*^*fl/fl*^ mice exposed to hyperglycemic conditions (Fig. 3A). TdTomato-labeled astrocytes were isolated from both normoglycemic and hyperglycemic mouse brain using a cell sorter (Fig. 3B; Additional file 1: Fig. S3A) and were subsequently validated through the expression of cell-specific marker genes (*aldh1l1, aqp4*, and *slc1a2* for astrocytes, as well as markers for other cell types including oligodendrocytes, microglia, neurons, and endothelial cells) (Additional file 1: Fig. S3B).

We then examined the differentially expressed genes (DEGs) and enriched gene ontologies in mouse brain astrocytes under hyperglycemic conditions. The principal component analysis (PCA), exhibiting the distribution of each sample, confirmed distinct transcriptional variances between vehicle-treated and STZ-treated astrocytes (Fig. 3C). A total of 234 DEGs were found between the two groups, with 146 genes upregulated and 88 genes downregulated in astrocytes from STZ-treated mouse brains (Fig. 3D). Gene ontology (GO) enrichment analysis of these 234 DEGs revealed diverse biological processes, including the regulation of response to stimulus, cell proliferation, and immune system process (Fig. 3D). Notably, most of genes associated with positive regulation of cell proliferation and inflammatory responses were significantly upregulated in STZ-treated astrocytes (Fig. 3E; Additional file 1: Fig. S4A, B). Furthermore, proinflammatory and neurotoxic genes, such as *Pla2g3*, *Fkbp5*, *Cxcl2*, and *Tnf* exhibited a marked increase in expression in hyperglycemic astrocytes compared to the control cells (Fig. 3F-H). However, neuroprotective genes crucial for maintaining brain homeostasis, including *Apln* and *IGF-1*, displayed a significant downregulation in astrocytes under hyperglycemic conditions (Fig. 3F). Additionally, GSEA analysis revealed an upregulation of genes associated with chemokine production and neutrophil or monocyte chemotaxis (Fig. 3I-K), along with a downregulation of genes related to brain homeostasis, such as nervous system regulation or synapse assembly (Fig. 3L; Additional file 1: Fig. S4C-E) in response to hyperglycemic conditions. These observations underscore that STZ-induced hyperglycemia can lead to the reprogramming of astrocytes into proinflammatory phenotypes.

To further determine the hyperglycemia-induced phenotypic shift of astrocytes, we profiled expression patterns of astrocyte under hyperglycemic conditions in terms of A1 reactive and A2 reactive genes in accordance with the previous study [30, 31]. Of notice, the majority of pan-reactive and A1 reactive genes were upregulated in astrocytes of hyperglycemic group, while there were less significant changes in A2 reactive genes in response to hyperglycemic conditions (Fig. 3M). This analysis revealed that STZ-induced hyperglycemia drives a reprogramming of astrocytes into neuroinflammatory A1 reactive phenotype.

### Hyperglycemia enhances the sensitivity to peripheral LPS-induced brain inflammation

**Fig. 4:**
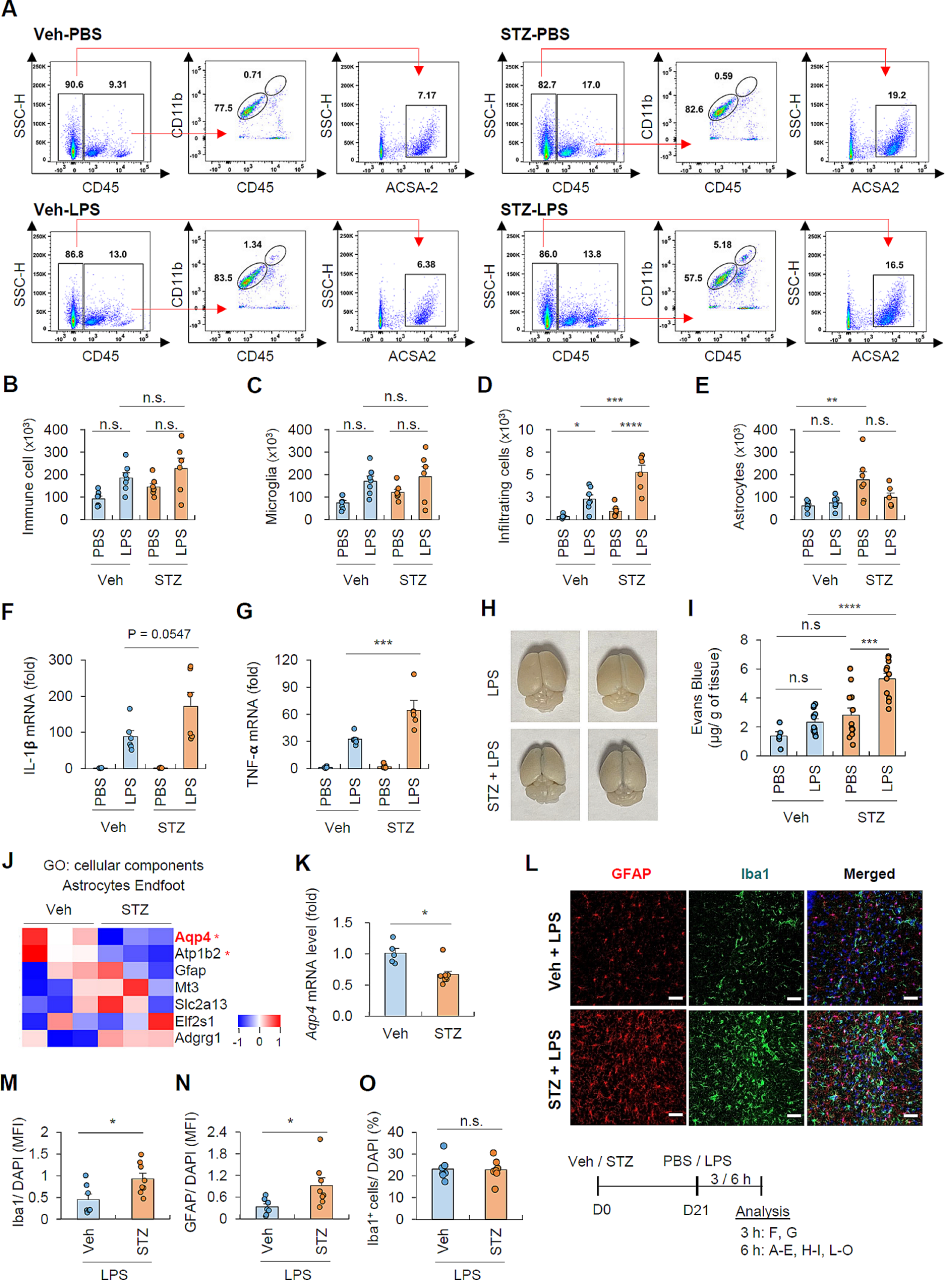
Hyperglycemic condition increases the vulnerability of brain to peripheral inflammatory stimulation (**A**) Representative flow cytometric analysis of brain cell populations in vehicle- and STZ-treated mice followed by an intraperitoneal injection of LPS (0.5 mg/kg, 6 h). (**B**-**E**) Quantification of each cell population in (**A**). (6 mice, STZ-LPS; 7 mice, other groups) (**F**-**G**) Quantification of mRNA levels of IL-1𝝱 and TNF-𝝰 in the brain extracts of veh- or STZ-treated mice upon PBS or LPS injection (0.5 mg/kg, 3 h). (5 mice, veh-PBS; 6 mice, veh-LPS, STZ-LPS; 8 mice, STZ-PBS) (**H**-**I**) Representative brain images (**H**) and quantification (**I**) of relative Evans blue extravasation into brain of veh- or STZ-treated mice upon LPS administration (0.5 mg/kg, 6 h). Evans blue was intravenously injected one hour before sacrifice. (5 mice, veh-PBS; 13 mice, veh-LPS; 12 mice, STZ-PBS; 11 mice, STZ-LPS) (**J**) Heatmap of astrocytes endfoot genes (GO:0097450) in RNA-seq data from veh- or STZ-treated mice. Asterisk indicates genes of FDR adjusted *p*-value < 0.05. (**K**) Quantification of mRNA level of *Aqp4* (aquaporin 4) in the brain extracts of veh- or STZ-treated mice. (5 mice, vehicle; 8 mice, STZ group) (**L**) Representative immunohistochemical images of hippocampal region of veh- or STZ- treated mice followed by LPS administration (0.5 mg/kg, 6 h). The coronal brain sections were stained with anti-GFAP (red) and anti-Iba1 (green). DAPI represents nuclear signal (blue). Scale bars = 50 μm. (**M**-**O**) Quantification of mean fluorescence intensity of Iba1 (**M**) or GFAP (**N**), and the number of Iba1 cells (**O**) per DAPI. (7 mice, veh-LPS; 8 mice, STZ-LPS) Data are presented as means ± SEM. Asterisks indicate significant differences between the groups as determined by two-way ANOVA with Bonferroni post hoc test (**B**-**G** and **I**) or Student’s two-tailed, unpaired *t* test (K and M-O); *P < 0.05, **P < 0.01, ***P < 0.001, ****P < 0.0001, n.s. not significant

As transcriptomic analysis exhibited a notable increase in chemokines or chemotaxis-associated genes in astrocytes under hyperglycemic conditions, we examined the infiltration of circulating myeloid cells, such as neutrophils or monocytes, into the brain in the presence of mild intraperitoneal lipopolysaccharide (LPS) stimulation (Fig. 4A). Systemic LPS administration did not induce a significant increase in the total immune cells (CD45^+^) and brain-resident microglia (CD45^med^ CD11b^med^) in the brains of both control and STZ-treated mice (Fig. 4B, C). However, LPS treatment alone significantly elevated the infiltration of circulating myeloid cells (CD45^hi^ CD11b^hi^) into the brain, and this LPS-triggered myeloid cell infiltration was markedly enhanced in hyperglycemic mice (Fig. 4D). These findings indicate that hyperglycemia enhances the recruitment of myeloid cells into the brain in the context of mild peripheral inflammation. Regarding astrocytes, the cell number of astrocytes was increased by STZ treatment as observed in Fig. 1G, but remained unaffected by LPS injection in both groups (Fig. 4E). To further assess the inflammatory status in the hyperglycemic brain, we examined the mRNA levels of proinflammatory cytokines. Following LPS injection, we observed elevated expression levels of proinflammatory cytokines IL-1𝝱 and TNF-𝝰 in the brain (Fig. 4F-G), and the elevation in Tnf-𝝰 mRNA was significantly amplified under hyperglycemic conditions. However, STZ-induced elevation in IL-1𝝱 mRNA did not reach statistical significance (Fig. 4F, *P* = 0.0547).

Along with the increased myeloid cell infiltration in hyperglycemic condition, we assessed the blood-brain barrier’s (BBB) integrity by examining the leakage of Evans Blue dye. Interestingly, STZ treatment alone did not induce a significant increase in BBB permeability, but significantly potentiated the disruption of the BBB induced by peripheral LPS (Fig. 4H-I). In line with these observations, RNA-seq analysis revealed that STZ treatment led to a decrease in the expression levels of astrocytic endfoot genes including aquaporin 4 (*Aqp4*) (Fig. 4J). The reduced expression of *Aqp4* in hyperglycemia was further confirmed in mouse brain tissue (Fig. 4K), suggesting that hyperglycemia may disrupt the integrity of astrocyte endfeet, which are crucial for maintaining the homeostatic function of the BBB.

To further investigate the impact of hyperglycemia on the inflammatory status of the brain, we conducted immunohistochemical analysis to assess gliosis in the hippocampal region. In the presence of peripheral LPS stimulation, STZ treatment resulted in a significant increase in the fluorescence intensity of Iba1^+^ and GFAP^+^ cells in the brain hippocampus (Fig. 4L-N; Additional file 1: Fig. S5). Notably, STZ-induced hyperglycemia did not affect the number of microglia in LPS-inflamed brains (Fig. 4O). These findings suggest that hyperglycemic conditions induce morphological changes in brain glial cells, leading to subsequent gliosis in response to mild peripheral inflammation.

### Hyperglycemia does not affect LPS-induced peripheral inflammation

**Fig. 5:**
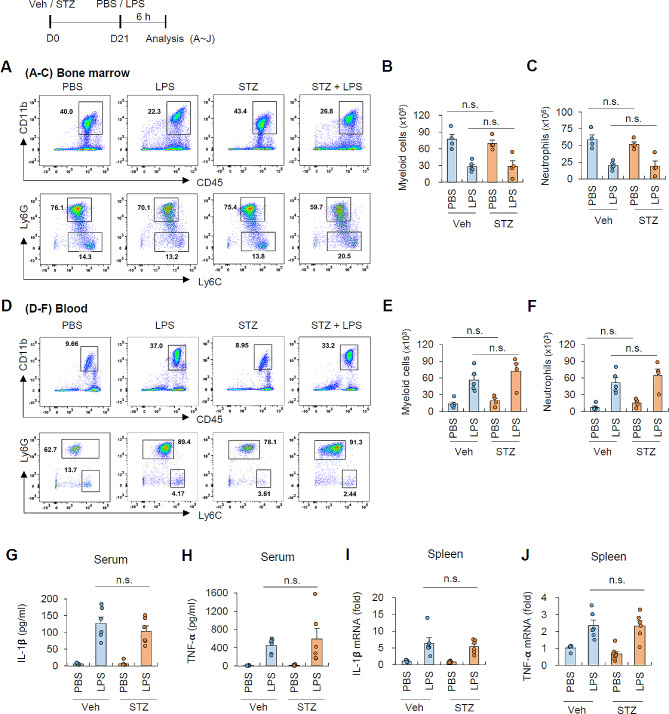
Hyperglycemic condition does not augment peripheral inflammation in response to systemic LPS administration (**A**-**C**) Flow cytometric analyses of bone marrow cells from vehicle- and STZ-injected mice followed by LPS injection (0.5 mg/kg, 6 h). (4 mice per group) (**D**-**F**) Flow cytometric analyses of circulating blood cells from vehicle- and STZ-injected mice followed by LPS injection (0.5 mg/kg, 6 h). (5 mice, vehicle groups; 4 mice, STZ groups) (**G**-**H**) Quantification of IL-1𝝱 (**G**) and TNF-𝝰 (**H**) protein level in the serum from vehicle- and STZ-injected mice followed by LPS injection (0.5 mg/kg, 6 h) using ELISA. (5 mice, veh-PBS; 6 mice, veh-LPS, STZ-LPS; 8 mice, STZ-PBS) (**I**-**J**) Quantification of mRNA levels of IL-1𝝱 (**I**) and TNF-𝝰 (**J**) from spleen extracts of vehicle- and STZ-injected mice followed by LPS injection (0.5 mg/kg, 6 h). (5 mice, veh-PBS; 6 mice, veh-LPS, STZ-LPS; 8 mice, STZ-PBS) Data are presented as means ± SEM. Significant differences between the groups were determined by two-way ANOVA with Bonferroni post hoc test. n.s. not significant

To explore how hyperglycemia can enhance the susceptibility to peripheral inflammation-induced neuroinflammation, we examined notable changes in the myeloid cell population in peripheral regions, including bone marrow and blood (Fig. S6). STZ treatment alone did not result in a significant alteration in the number of myeloid cells (CD45^+^CD11b^+^), monocytes (CD45^+^CD11b^+^Ly6C^+^), and neutrophils (CD45^+^CD11b^+^Ly6G^+^Ly6C^low^) in the bone marrow (Fig. 5A). Following intraperitoneal LPS injection, both control and STZ groups exhibited a decrease in the number of myeloid cells and neutrophils in the bone marrow, likely due to their egress from the bone marrow (Fig. 5B, C). Importantly, there was no significant difference in the number of bone marrow myeloid cells between control and hyperglycemic mice upon LPS administration (Fig. 5B, C). Similarly, STZ treatment alone did not cause significant changes in the number of myeloid cells and neutrophils in the circulating blood (Fig. 5D). While LPS treatment induced a substantial increase in circulating myeloid cells and neutrophils, there was no significant difference observed between control and hyperglycemic conditions (Fig. 5E, F).

We then examined the impact of hyperglycemia on the proinflammatory cytokine levels in the periphery. LPS administration led to a robust increase in IL-1β and TNF𝝰 production in both the serum (Fig. 5G, H) and spleen (Fig. 5I, J) of mice. However, the hyperglycemic condition did not affect this LPS-induced production of proinflammatory cytokines (Fig. 5G-J). These results collectively indicate that our hyperglycemic conditions do not alter the LPS-induced myeloid cells’ population and their immune responses in the periphery, suggesting that the heightened susceptibility of the brain to hyperglycemia is not likely attributed to increased peripheral inflammation.

### Hyperglycemia induces a specific increase in the responsiveness of brain microglia

**Fig. 6:**
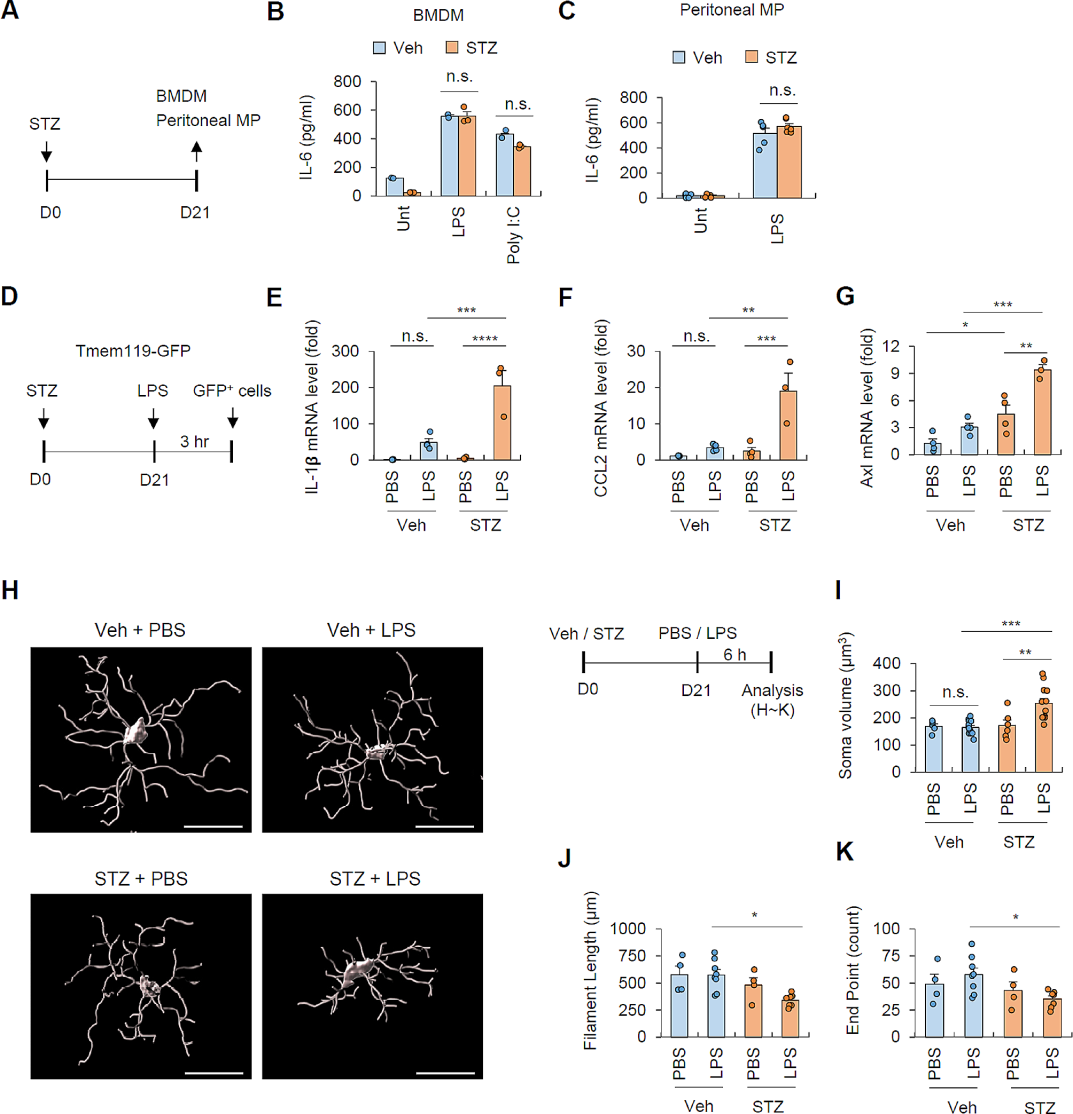
Hyperglycemic condition increases the sensitivity of microglia to systemic LPS administration (**A**) Experimental scheme of in vitro experiment using bone marrow-derived macrophages (BMDMs) and peritoneal macrophages (MP) isolated from normoglycemic (vehicle) and hyperglycemic (STZ-injected) mice. (**B**-**C**) Quantification of extracellular IL-6 levels from cultured BMDMs (B, 2 mice, vehicle; 3 mice, STZ) or peritoneal macrophages (C, 4 mice, vehicle; 5 mice, STZ) upon LPS (0.25 µg/ml, 3 h) or poly I: C (30 µg/ml, 3 h) treatment using ELISA. (**D**) Experimental scheme of microglia (GFP^+^) isolation from *Tmem119-EGFP* mice subjected to veh- or STZ-injection followed by LPS injection (0.5 mg/kg, 3 h). (**E**-**G**) Quantification of mRNA levels of IL-1𝝱 (E), CCL-2 (F) and Axl (G) from isolated microglia using real-time qPCR. (*n* = 3, STZ-LPS group; *n* = 4, other groups; each RNA sample was pooled from 5-independent mice) (H) Representative images of morphological analysis of microglia in veh- and STZ- injected mice upon LPS administration (0.5 mg/kg, 6 h) using Imaris software. (**I**-**K**) Quantification of soma volume (**I**), filament length (**J**), and end point counts (**K**) of microglia. (I, 5 mice, veh-PBS; 13 mice, veh-LPS; 6 mice, STZ-PBS; 12 mice, STZ-LPS; J-K, 4 mice, veh-PBS, STZ-PBS; 8 mice, veh-LPS; 7 mice, STZ-LPS) Three or four cells were randomly selected and averaged for each mouse brain section. Data are presented as means ± SEM. Asterisks indicate significant differences between the groups as determined by two-way ANOVA with Bonferroni post hoc test (B-C, E-G and I-K) *P < 0.05, **P < 0.01, ***P < 0.001, ****P < 0.0001, n.s. not significant

To explore the significant influence of hyperglycemia on the responsiveness of peripheral macrophages, we isolated bone marrow cells from both control and STZ-treated mice and induced their differentiation into bone marrow-derived macrophages (BMDMs). Additionally, we isolated peritoneal macrophages from peritoneal lavage collected from control and STZ mice (Fig. 5A). Subsequently, we analyzed in vitro IL-6 production from these macrophages following stimulation with poly(I: C) or LPS. In BMDMs, STZ-induced hyperglycemia did not alter the IL-6 production induced by LPS or poly(I: C) (Fig. 6B).Similarly, the level of LPS-induced IL-6 production in peritoneal macrophages was not affected by STZ-induced hyperglycemia (Fig. 6C). These results indicate that the sensitivity of peripheral macrophages to toll-like receptor signaling remained unchanged under hyperglycemic conditions.

Then, to examine potential alterations in the responsiveness of brain microglia following STZ treatment, we used *Tmem119-EGFP* mice, where microglia are specifically labeled with GFP. *Tmem119-EGFP* mice were administered with STZ, and following a 21-day interval, they received an intraperitoneal injection of LPS (Fig. 6D). Brain microglia were then isolated based on GFP fluorescence (Additional file 1: Fig. S7) and examined for the expression of inflammation-related genes. Notably, we observed a significant increase in the expression of inflammatory genes such as IL-1β and CCL2 in microglia from STZ-treated mice compared to microglia from control mice, following peripheral LPS injection (Fig. 6E, F). Additionally, the expression level of *Axl*, a representative gene associated with dysfunctional or neurodegenerative microglia [32], was also elevated in the STZ-treated microglia, regardless of LPS stimulation (Fig. 6G).

To further characterize the phenotypic changes of microglia induced by hyperglycemia, we examined the morphology of microglia under normal and hyperglycemic conditions (Fig. 6H). Consequently, STZ treatment led to a significant increase in the soma volume and a decrease in the length and the number of endpoints of microglia in the presence of LPS stimulation (Fig. 6I-K; Additional file 1: Fig. S8). These morphological analyses demonstrate that peripheral hyperglycemia induces a phenotypic shift in brain microglia towards an enhanced innate immune-active state in the context of peripheral LPS stimulation.

### High extracellular glucose induces Glut1-dependent phenotypic changes in primary astrocyte culture

**Fig. 7:**
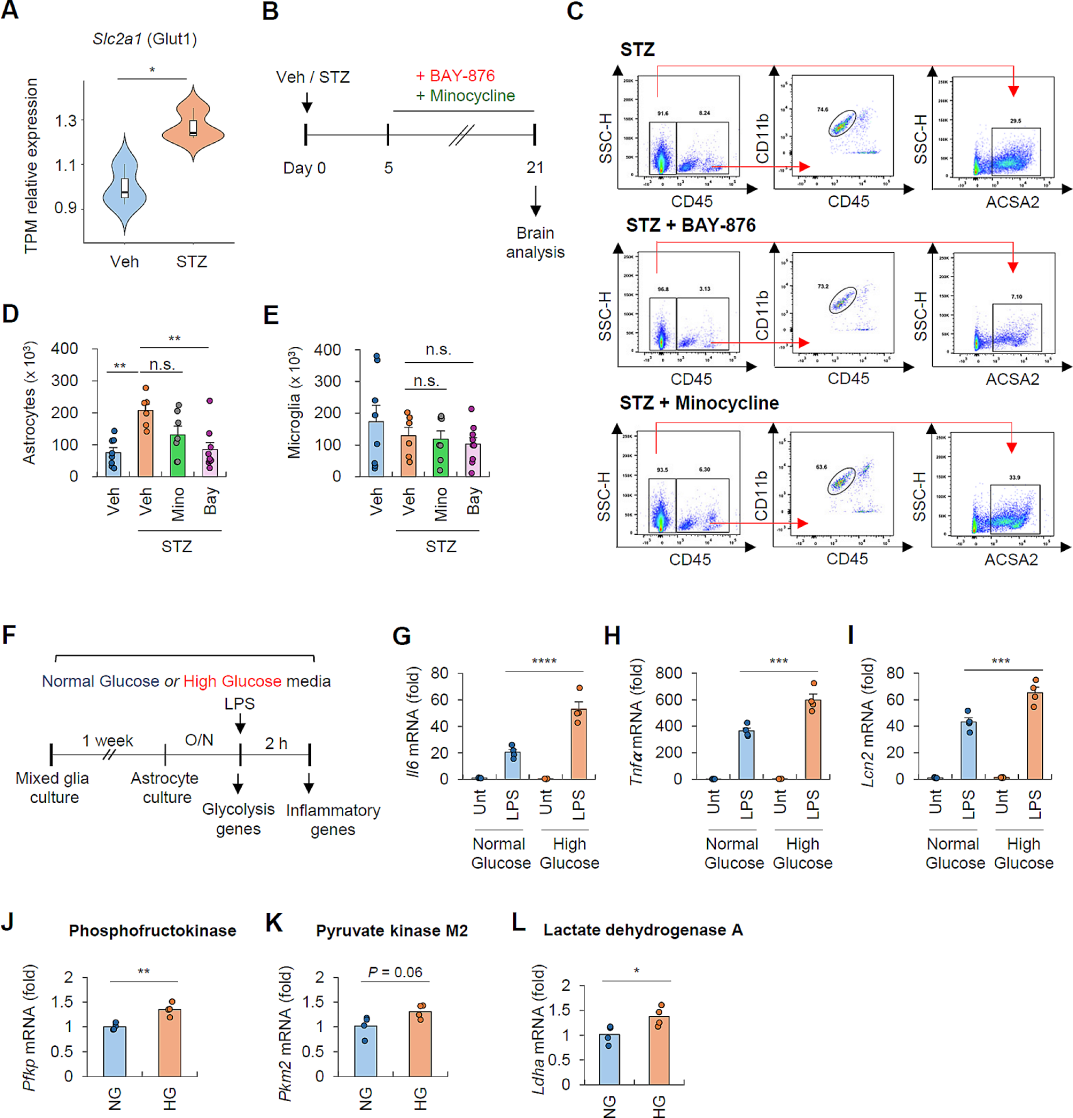
High extracellular glucose increases the expression of glycolytic and proinflammatory molecules in astrocytes. (**A**) The relative expression level (fold change) of *slc2a1* in hyperglycemic (STZ-treated) astrocytes in relation to normoglycemic astrocytes based on TPM read counts obtained from bulk RNA-seq data. (**B**) Experimental scheme of BAY-876 and minocycline administration in normoglycemic or hyperglycemic mice. Mice were given daily intravenous injections of BAY-876 (1.0 mg/kg) or minocycline (45 mg/kg) from day 5 to day 21. (**C**) Representative flow cytometric analysis of brain cell populations in STZ-treated mice followed by vehicle, BAY-876, or minocycline injection. (**D**-**E**) Quantification of astrocytes (**D**) or microglia (**E**) population in (**C**). (8 mice, vehicle; 6 mice, STZ; 7 mice, STZ + minocycline; 9 mice, STZ + BAY-876 group) (F) Experimental scheme of in vitro primary mixed glial cells and astrocytes cultured in normal glucose (5.5 mM) or high glucose (25 mM) DMEM, followed by LPS treatment (0.2 µg/ml, 2 h) (**G**-**I**) Quantification of mRNA levels of *Il6* (**G**), *Tnf*a (**H**), and *Lcn2* (**I**) from primary astrocytes cultured in normal or high glucose-DMEM following LPS stimulation (*n* = 4 for all groups). (**J**-**L**) Quantification of mRNA levels of glycolytic genes of primary astrocytes cultured in normal or high glucose DMEM (*n* = 4 for all groups). Data are presented as means ± SEM. Asterisks indicate significant differences in one-way ANOVA with Bonferroni post hoc test (D-E), two-way ANOVA with Bonferroni post hoc test (**G**-**I**), and Student’s two-tailed, unpaired *t* test (**J**-**L**). *P < 0.05, **P < 0.01, ***P < 0.001, ****P < 0.0001, n.s. not significant

To explore how systemic hyperglycemic conditions drive brain astrocyte proliferation, we checked the potent role of glucose transporter Glut1. Of interest, RNA-seq analysis revealed that STZ treatment significantly increased *Slc2a1* mRNA expression in astrocyte (Fig. 7A). Then, we assessed STZ-induced astrocyte proliferation in the presence of Glut1 inhibitor BAY-876 (Fig. 7B). In addition, we also administered minocycline, commonly used to inhibit microglial activation, into STZ-treated mice to examine the effect of microglia on the astrocyte proliferation. Consequently, STZ-induced increase in astrocyte population was significantly reduced by BAY-876, but not by minocycline (Fig. 7C, D). In contrast, both chemicals had no effect on the number of microglia (Fig. 7E). These observations indicate that STZ-induced hyperglycemic conditions modulate astrocyte proliferation in a Glut1-dependent manner.

To further confirm the glucose-driven reprogramming of astrocytes, primary mouse astrocytes were cultured in normal or high glucose medium for one week (Fig. 7F). Then, we checked the responsiveness of astrocytes to LPS stimulation. Notably, high glucose medium led to an enhanced production of proinflammatory and reactive genes in astrocytes following LPS treatment (Fig. 7G-I). Given that hyperglycemia induces training of macrophages via glycolysis-dependent epigenetic modifications [33], we examined the expression levels of glycolytic enzymes in astrocytes under normal or high glucose medium. Intriguingly, high glucose medium-exposed astrocytes exhibited an increased expression of glycolytic enzymes compared to astrocytes in normal glucose environments (Fig. 7J-L). These findings suggest that high extracellular glucose can drive astrocyte reprogramming via inducing glycolysis-dependent alterations of chromatin accessibility of proinflammatory genes.

### Hyperglycemia potentiates peripheral LPS-induced cognitive impairment

**Fig. 8:**
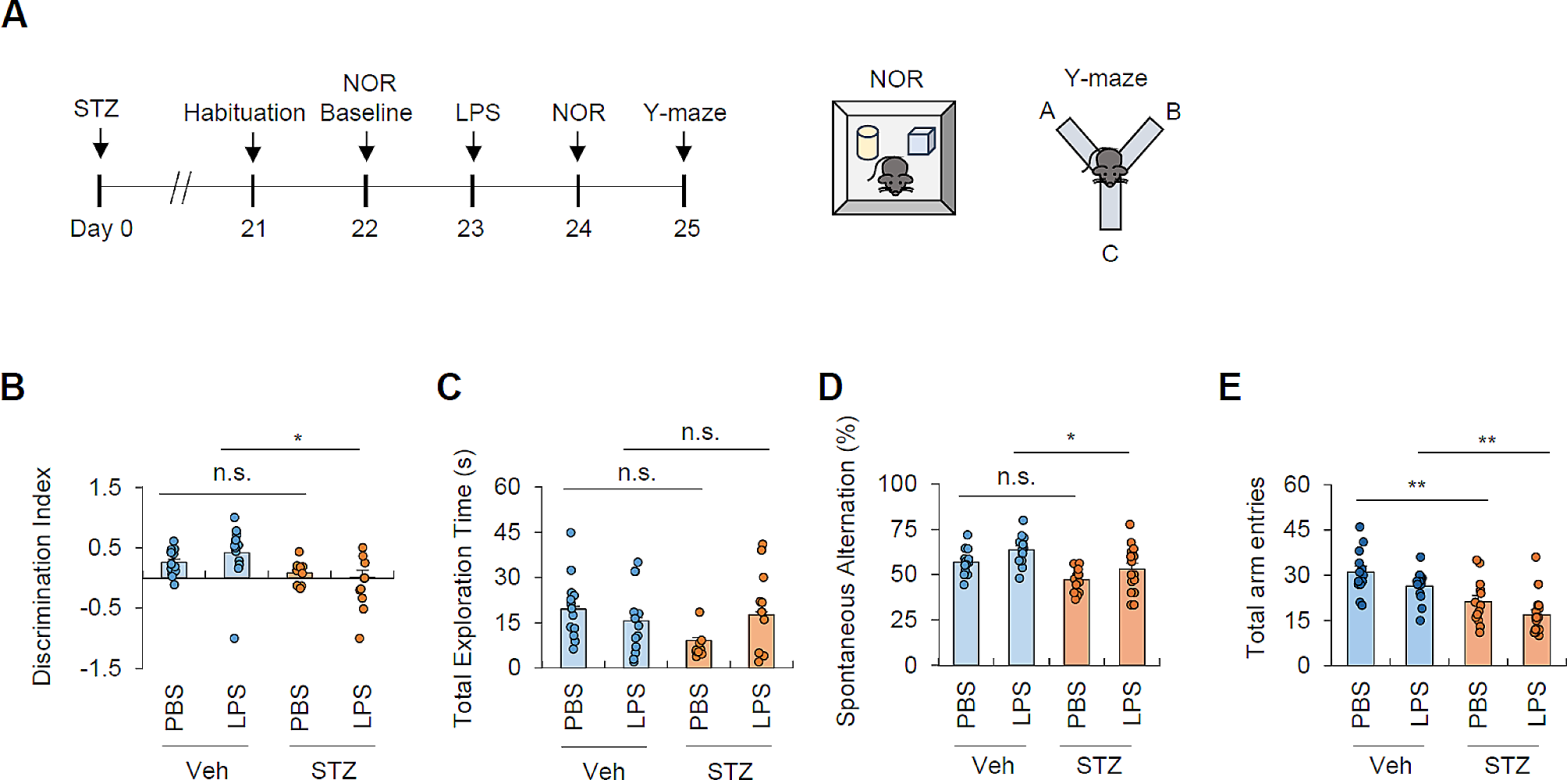
Hyperglycemic conditions impair cognitive function upon mild peripheral LPS stimulation (**A**) Experimental scheme for Novel object recognition (NOR) and Y-maze behavioral tests. Control and STZ-treated mice were intraperitoneally administered with LPS (0.25 mg/kg) and rested for 24 h before the tasks. (B-C) NOR task conducted for vehicle- and STZ- treated mice followed by PBS or LPS injection. (14 mice, veh-PBS; 13 mice, all other groups) The discrimination index calculation (**B**) and total exploration measurement (**C**) were shown. Mice that did not explore both objects during the training phase were excluded from the analysis. (D-E) Y-maze task conducted for vehicle- and STZ- treated mice followed by PBS or LPS treatment. The spontaneous alternation calculation (**D**) and total arm entries count (**E**) were shown. (13 mice, veh-PBS; 14 mice, veh-LPS; 14 mice, STZ-PBS; 16 mice, STZ-LPS group) Mice with total arm entries fewer than 10 were excluded from the calculation. Data are presented as means ± SEM. Asterisks indicate significant differences between the groups as calculated by two-way ANOVA with Bonferroni post hoc test. *P < 0.05, **P < 0.01, n.s. not significant

To determine the impact of hyperglycemia on the brain functions in the context of inflammatory stimuli, we conducted behavioral tests to assess the cognitive functions (Fig. 8A). Of note, novel object recognition (NOR) task demonstrated that mild LPS administration significantly impaired cognitive functions of hyperglycemic mice compared with control mice, whereas hyperglycemia alone did not cause baseline cognitive impairment of mice (Fig. 8B). Additionally, there were no significant differences in total exploration time between the two groups upon LPS injection, implying the locomotor function was not affected in hyperglycemic mice (Fig. 8C). Similarly, Y-maze task revealed a reduced spontaneous alternation and total arm entries in hyperglycemic mice upon LPS injection compared with the control mice (Fig. 8D, E), suggesting that hyperglycemic condition can impair brain performances upon a mild inflammatory stimulus. In the vehicle- and PBS-treated group, both the discrimination index and spontaneous alternations significantly diverged from their respective theoretical mean values of 0 (no discrimination) and 50% (random behavior), substantiating the biological relevance of the cognitive evaluation (Additional file 1: Fig. S9A-B).

The diminished total number of arm entries also indicates a decreased willingness of hyperglycemic mice to explore unfamiliar environments. Collectively, these results demonstrate that hyperglycemia can enhance the brain’s vulnerability to mild peripheral inflammatory stimulation, ultimately impairing its function through a mechanism involving hyperglycemia-induced alterations of astrocytes.

## Discussion

**Fig. 9:**
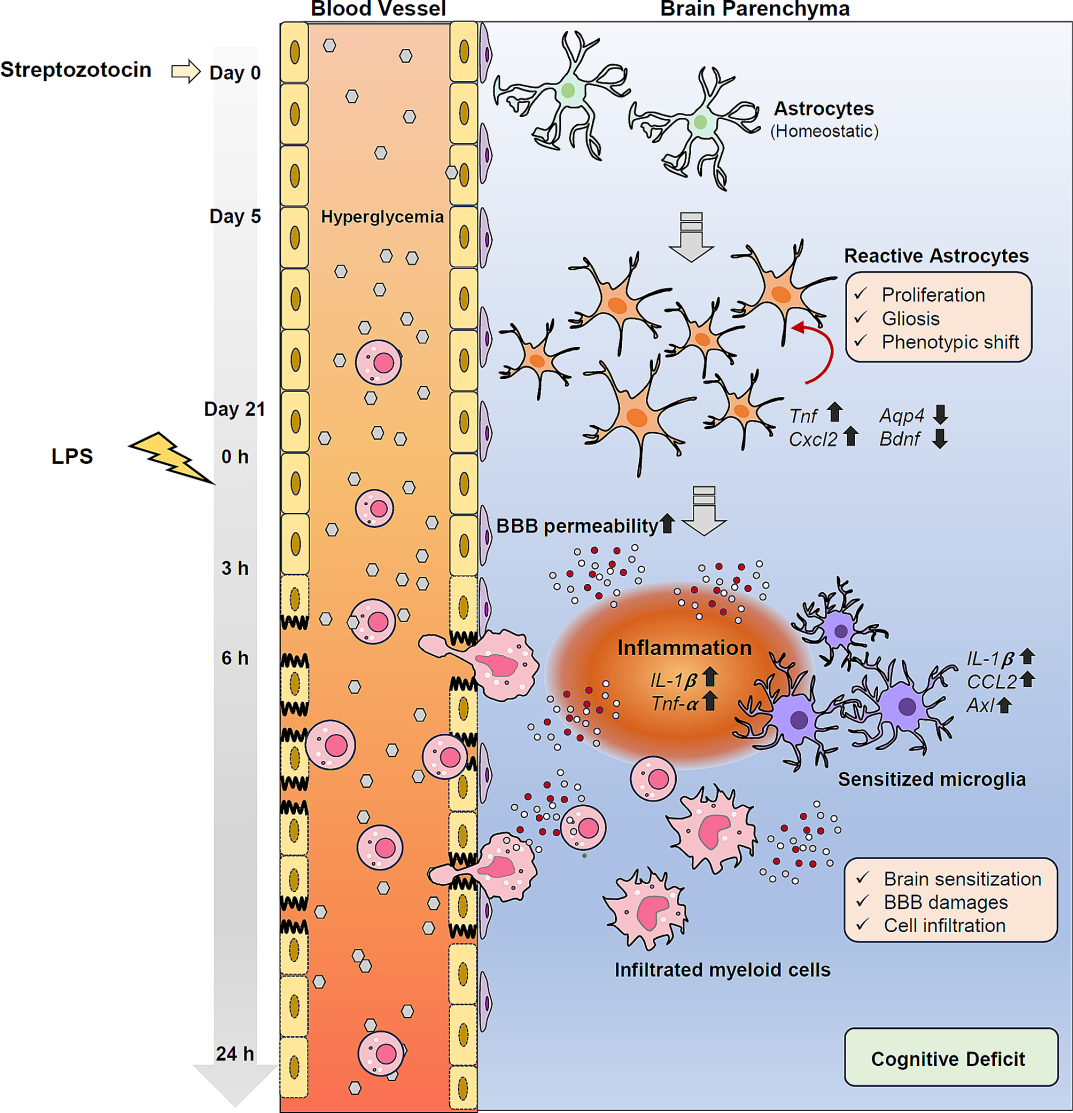
Proposed mechanisms for how hyperglycemia amplifies neuroinflammation sensitivity. Hyperglycemic conditions induced by streptozotocin promote astrocyte proliferation and reprogram homeostatic astrocytes into inflammatory phenotype in brains. Upon peripheral inflammation induced by lipopolysaccharide (LPS), reactive astrocytes augment LPS-induced blood-brain barrier (BBB) damages and myeloid cell infiltration into brains, which lead to neuroinflammation and the subsequent cognitive dysfunction

Diabetic condition poses a risk of severe complications and often worsens existing brain pathologies. Nevertheless, the direct impact of hyperglycemic burden on brain cells and their contribution to the progression of brain diseases remain unclear. In the present study, we attempted to elucidate hyperglycemia-induced changes in brain glial cells using flow cytometric and immunohistochemical analyses. In particular, we conducted an RNA-sequencing-based transcriptomic analysis of brain astrocytes under hyperglycemic stress. Our findings revealed that hyperglycemia stimulates the proliferation of astrocytes, the central regulators of brain glucose and energy metabolism, leading to the reprogramming of astrocytes. Furthermore, our results demonstrate that the hyperglycemia-induced shift in astrocyte phenotype enhances the brain’s sensitivity to mild peripheral inflammation. (Fig. 9) This contrasts with most previous studies, which primarily focused on understanding the impact of hyperglycemia in acute stroke or reperfusion/ischemic conditions [34–37].

Astrocytes, the most abundant and diverse type of glial cells, play crucial roles in mediating a wide range of brain functions and immune regulation within the brain [38, 39]. Recent studies have revealed that proliferative astrocytes represent a distinct reactive subtype, differing from mature astrocytes, which remain quiescent and non-proliferative, and are observed in response to physical injuries such as traumatic brain injury or spinal cord injury [40–42]. These proliferating astrocytes can exert both protective and detrimental function in the brain [43]. For instance, in the traumatic brain injury or spinal cord injury, reactive astrocytes undergo proliferation and exhibit neuroprotective or scar-forming properties to impede the spread of inflammation [44, 45]. Conversely, astrocyte proliferation has also been linked to brain pathologies, such as neuroinflammation and gliosis [46, 47]. Therefore, the specific effects of proliferative astrocytes may vary depending on the circumstances, warranting further investigation for a comprehensive understanding. Our findings in this study demonstrate that astrocyte reprogramming induced by hyperglycemia involves heightened reactivity, neurotoxicity, and proliferation. In contrast to our observations, Li et al. proposed that hyperglycemia inhibits astrocyte proliferation [48]. However, it’s important to note that this study was conducted using in vitro astrocyte cultures exposed to elevated extracellular glucose levels. While the discrepancy might be influenced by differences in in vivo and in vitro observations, it remains crucial to verify the effects of high glucose levels on astrocytes in an in vivo setting.

Notably, our findings demonstrate that hyperglycemia facilitates the disruption of BBB in response to peripheral inflammatory stimulation. While astrocytic endfeet structure is crucial for maintaining BBB integrity [49], we also observed a downregulation of *Aqp4*, a pivotal gene of astrocytic endfeet, and an upregulation of chemotaxis-associated genes in astrocytes under hyperglycemic condition. These alterations in astrocytes may contribute to the increased permeability of the BBB in response to mild peripheral inflammation. Supporting these findings, the hyperglycemic condition significantly intensified the infiltration of myeloid cells into the brain triggered by peripheral LPS. Regarding the substantial impact of hyperglycemia on other BBB components, such as endothelial cells and pericytes, further investigations will be valuable to enhance our understanding.

Several previous studies have explored the effects of prolonged hyperglycemia on peripheral immune responses [50–53]. Notably, a recent investigation uncovered that hyperglycemia induces a trained immune response in peripheral myeloid cells, resulting in proinflammatory and pro-atherosclerotic phenotypes [33]. However, under our hyperglycemic conditions, we did not observe any changes in the reactivity of peripheral inflammatory cells. The discrepancy in previous studies may be attributed to their consideration of long-term hyperglycemic effects. Nevertheless, our relatively short-term hyperglycemic condition led to the reprogramming of astrocytes into a proinflammatory phenotype without impacting peripheral immune cells. Supporting these findings, our in vitro results from astrocyte cultures also demonstrated that a high-glucose medium induces the expression of glycolytic enzymes and increases the LPS responsiveness of astrocytes. It will be thus intriguing to examine the epigenetic modifications in astrocytes under hyperglycemic conditions. Consequently, our study sheds light on the crucial role of astrocytes in the context of hyperglycemia-mediated brain pathological conditions.

Concerning the direct influence of hyperglycemia on the brain, our observations reveal that hyperglycemia triggers both functional and morphological changes in microglia, alongside astrocytes. Notably, hyperglycemia significantly potentiates the innate immune potential of microglia to mild peripheral inflammation. This unexpected finding implies that hyperglycemia has the potential to reprogram both microglia and astrocytes. Since microglia are the major cell type engaged in brain immune responses and are substantially influenced by astrocytes [54], further transcriptomic and epigenetic analyses of microglia will be crucial to unravel the hyperglycemia-induced sensitization of brain glial cells to neuroinflammation.

In conclusion, the present study reveals the proliferation of reactive astrocytes exhibiting proinflammatory characteristics under hyperglycemic conditions. This transformation in astrocytes increases the susceptibility of brain immune responses, making it more vulnerable to mild peripheral stimulations. Given that we did not explore the function of candidate genes induced by hyperglycemia in astrocytes, further investigation is necessary to comprehend their functional relevance. Our findings highlight the potential roles of astrocytes in exacerbating diabetes-associated brain diseases, and the hyperglycemia-induced alterations in astrocytes could serve as a therapeutic target to alleviate brain pathologies associated with diabetic complications.

### Electronic supplementary material

Below is the link to the electronic supplementary material.

Supplementary Material 1

## Data Availability

All data needed to evaluate the conclusions in the paper are present in the paper or the Supplementary Materials. RNA-Seq data are available through the National Center for Biotechnology Information Gene Expression Omnibus. (GSE247033)
