# Supplementary material for: Hyperglycemia enhances brain susceptibility to lipopolysaccharide-induced neuroinflammation via astrocyte reprogramming
Source: J Neuroinflammation. 2024 May 27;21:137. doi: 10.1186/s12974-024-03136-1 (PMC11131277; doi:10.1186/s12974-024-03136-1)
Supplement: Supplementary file 1 — Supplementary Material 1 [file 12974_2024_3136_MOESM1_ESM.pdf]

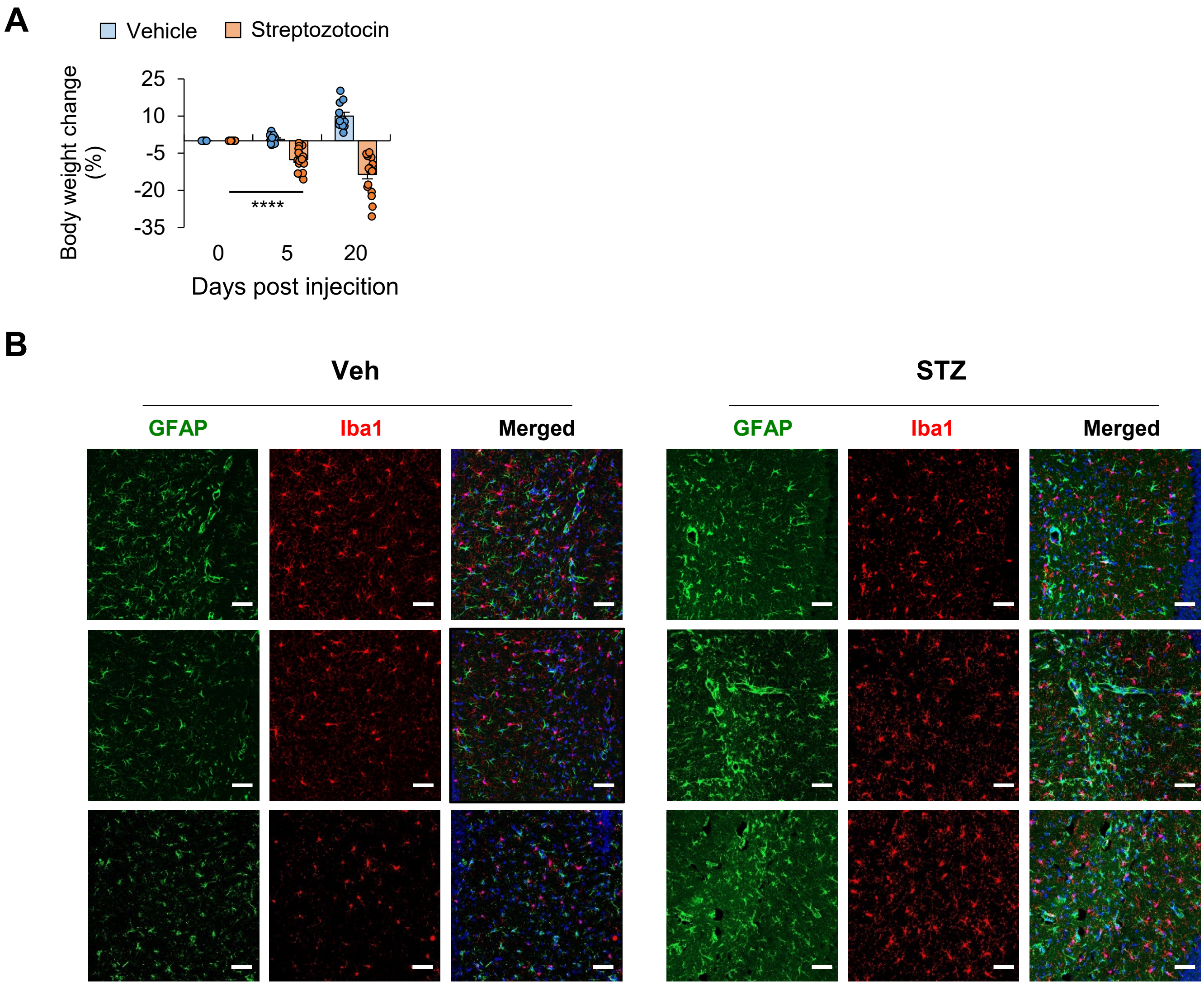

Supplementary Figure S1. Flow cytometric and immunohistochemical analyses of mouse brain from normoglycemic and STZ-induced hyperglycemic condition. (A) Body weight measurement after STZ injection in wild type mice (14 mice, vehicle; 18 mice, STZ group) (B) Immunohistochemical images of hippocampal regions of brain coronal sections; astrocytes and microglia are stained with anti-GFAP (green) and anti-Iba1 (red), respectively. Scale bars = 50 μm. Data are presented as means ± SEM. Asterisks indicate significant differences in one-way ANOVA with Bonferroni post hoc test. ****$P < 0.0001$.

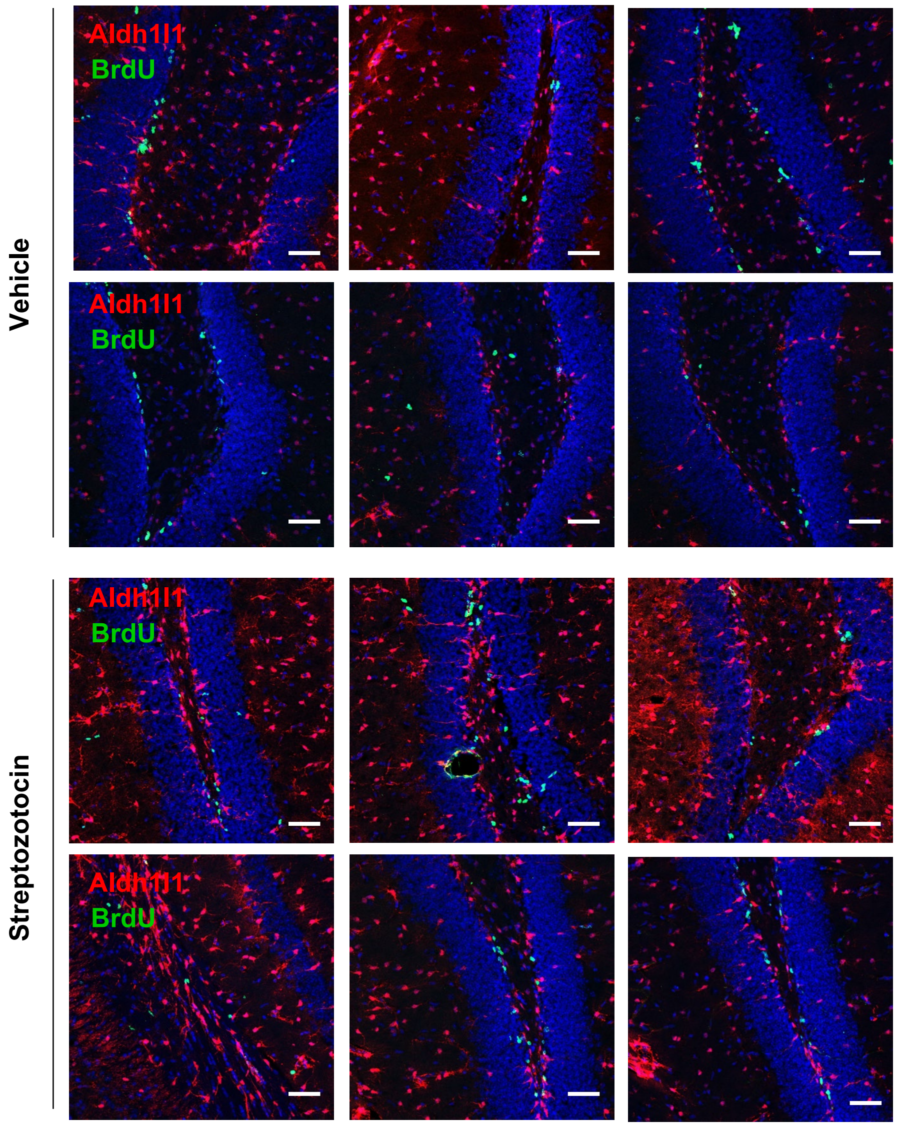

Supplementary Figure S2. BrdU^{+} astrocytes indicate astrocyte proliferation in hyperglycemic condition. Immunohistochemical images depicting the colocalization of astrocytes (tdTomato) and BrdU (green) in brain hippocampal region to label proliferating astrocytes. DAPI (blue) indicates nuclei signaling. Scale bars = 50 µm.

A

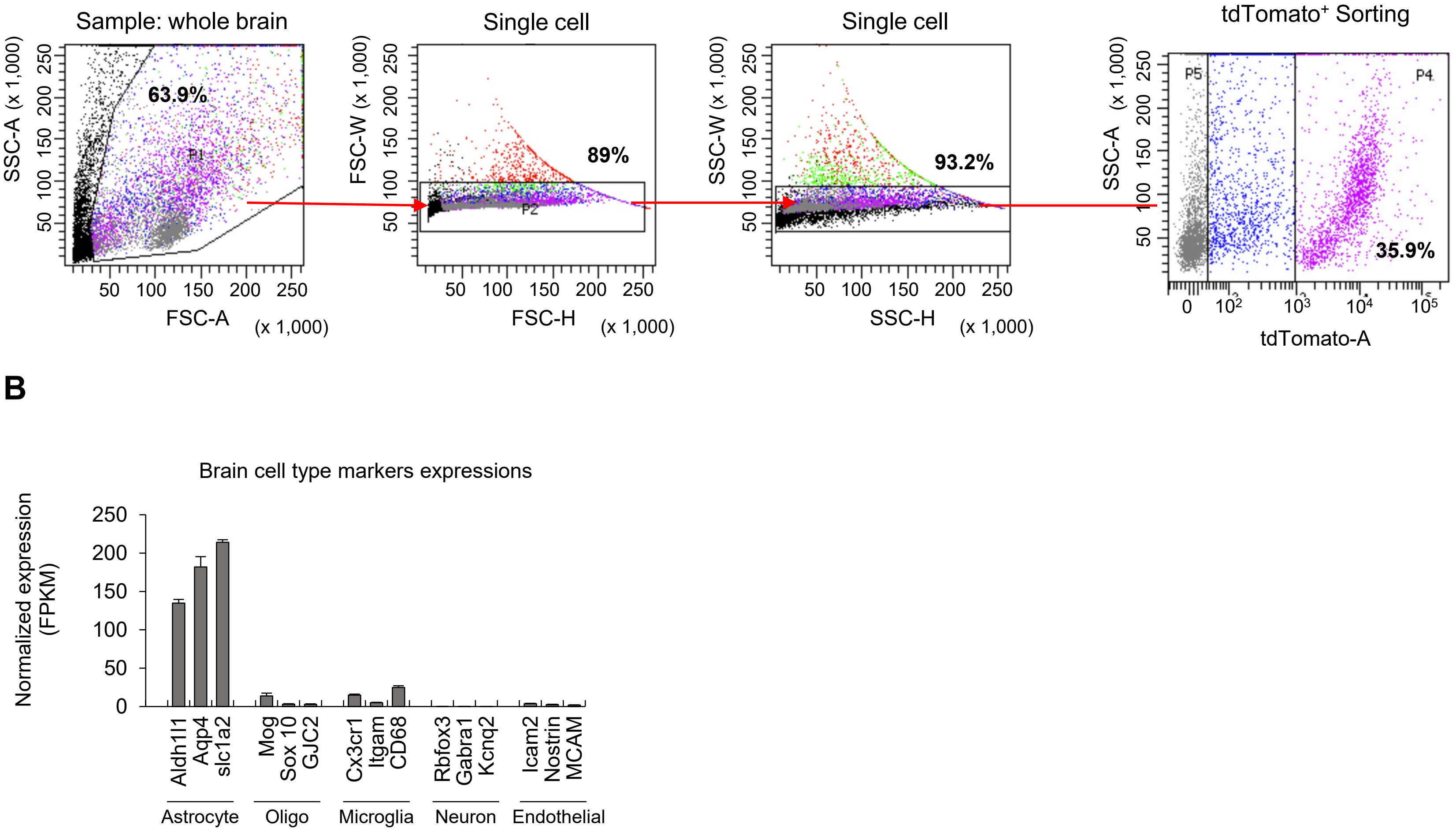

Supplementary Figure S3. RNA-seq analysis exhibits transcriptional differences between normoglycemic and hyperglycemic astrocytes. (A) Representative gating strategies of cell sorting of astrocytes (tdTomato+ cells) from brain single cell suspensions of tdTomato^{flfl};Aldh1l1-CreERT mice. (B) Validation of astrocytes using gene markers of astrocytes, oligodendrocytes, microglia, neuron, and endothelial cells; FPKM= Fragments Per Kilobase of transcripts per Million mapped reads.

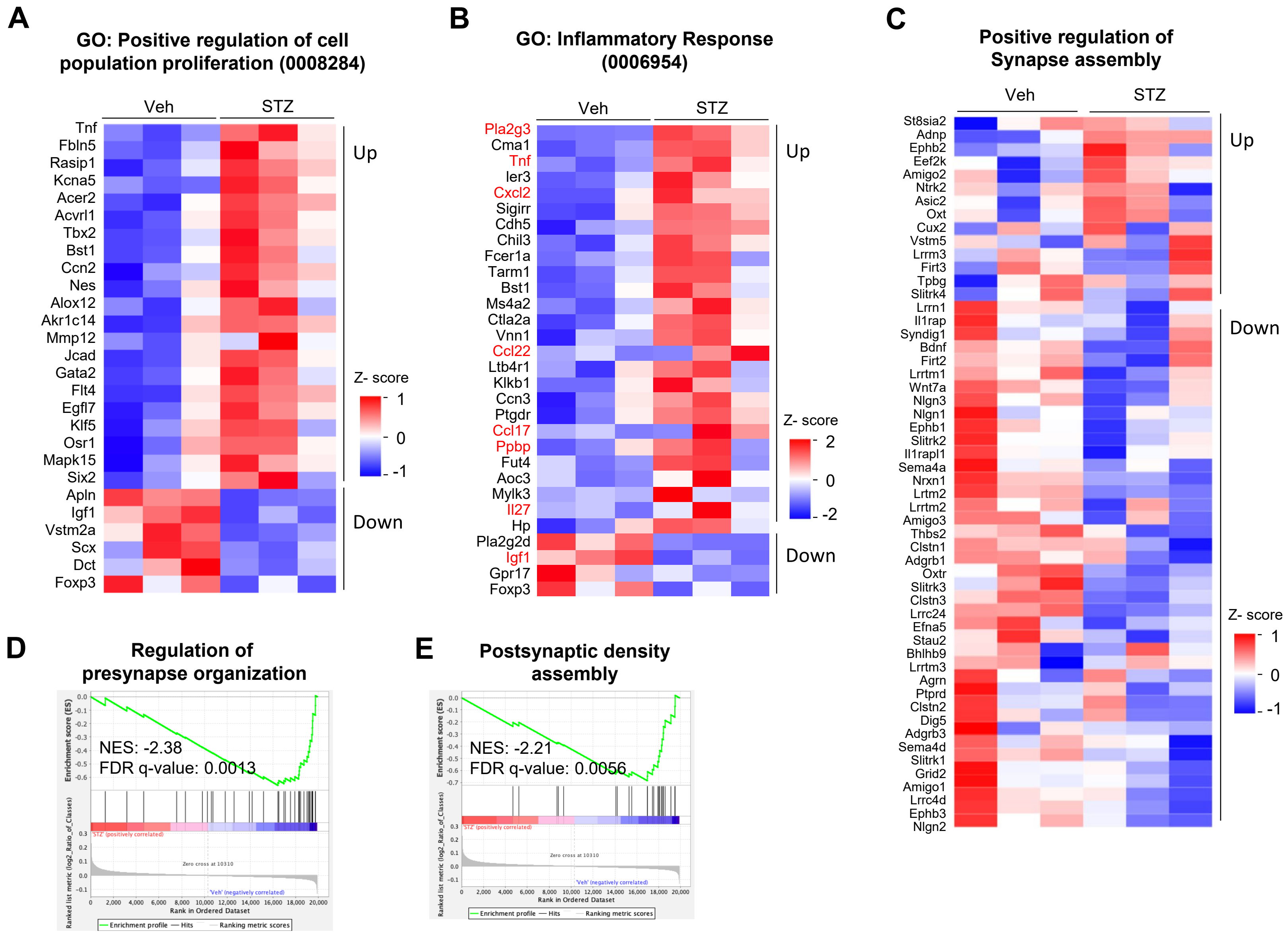

Supplementary Figure S4. Gene ontology analysis of hyperglycemic astrocytes compared with normoglycemic astrocytes. (A-B) Heatmap representation of Gene Ontology (GO) analysis of GO:0008284 (positive regulation of cell population proliferation) (A) and GO: 0006954 (inflammatory responses) (B) with colors indicating Z scores; adjusted p-value < 0.05, |fold change| > 1.5. The heatmap is in the order of increasing p-values. (C) Heatmap analysis of genes associated with positive regulation of synapse assembly of the whole transcriptomes with colors indicating Z scores. (D-E) GSEA analysis of GO terms of regulation of presynapse organization (D) and postsynaptic density assembly (E).

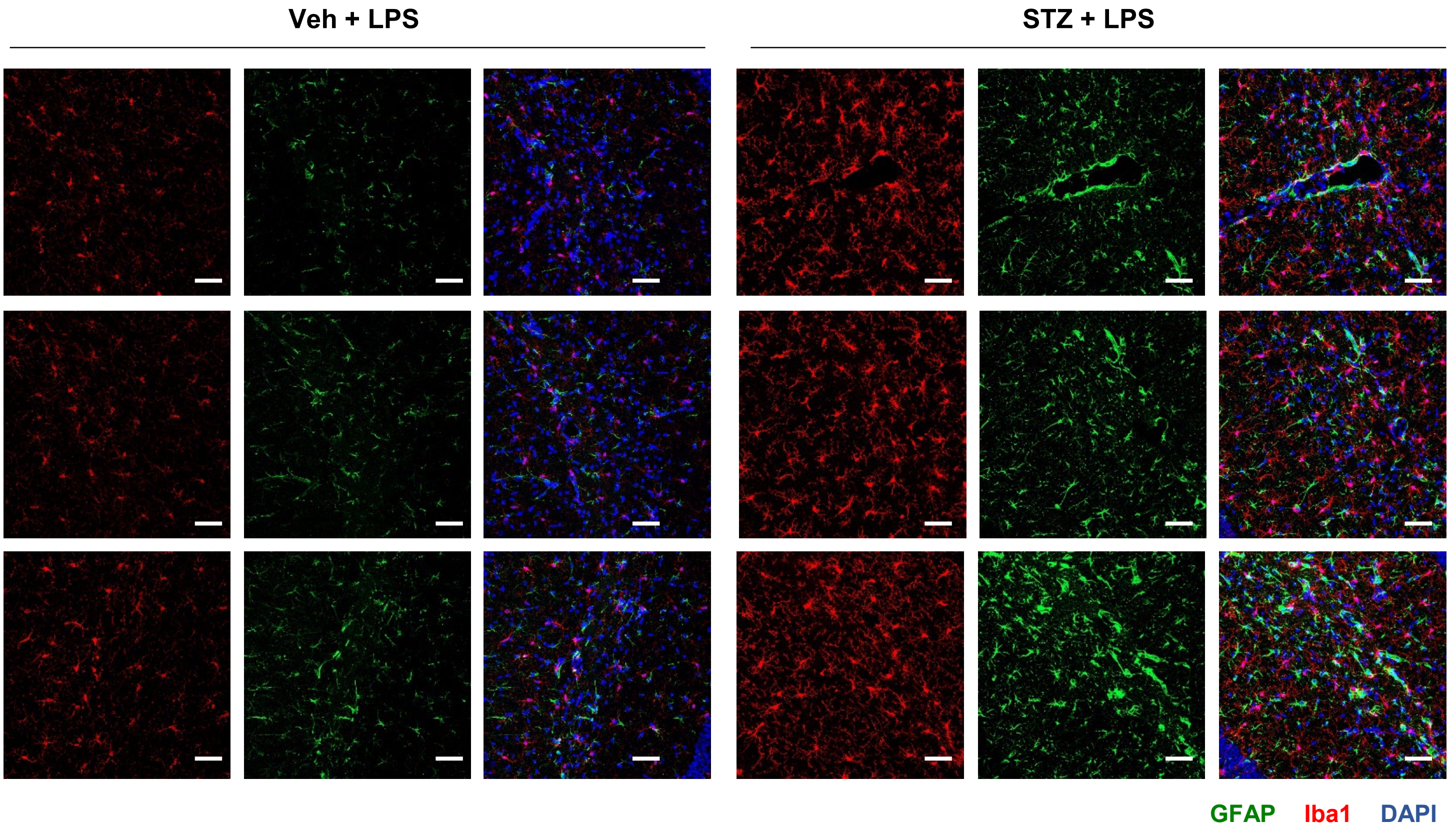

Supplementary Figure S5. Hyperglycemia induces a morphological change in brain glial cells upon systemic LPS injection. Immunohistochemical images of the hippocampal regions of vehicle- and STZ-treated mice upon LPS administration (0.5 mg/kg; 6 h). Astrocytes and microglial gliosis are evaluated using anti-GFAP (green) and anti-Iba (red) antibodies, respectively. DAPI represents the nuclei signaling. Scale bars= 50 μm.

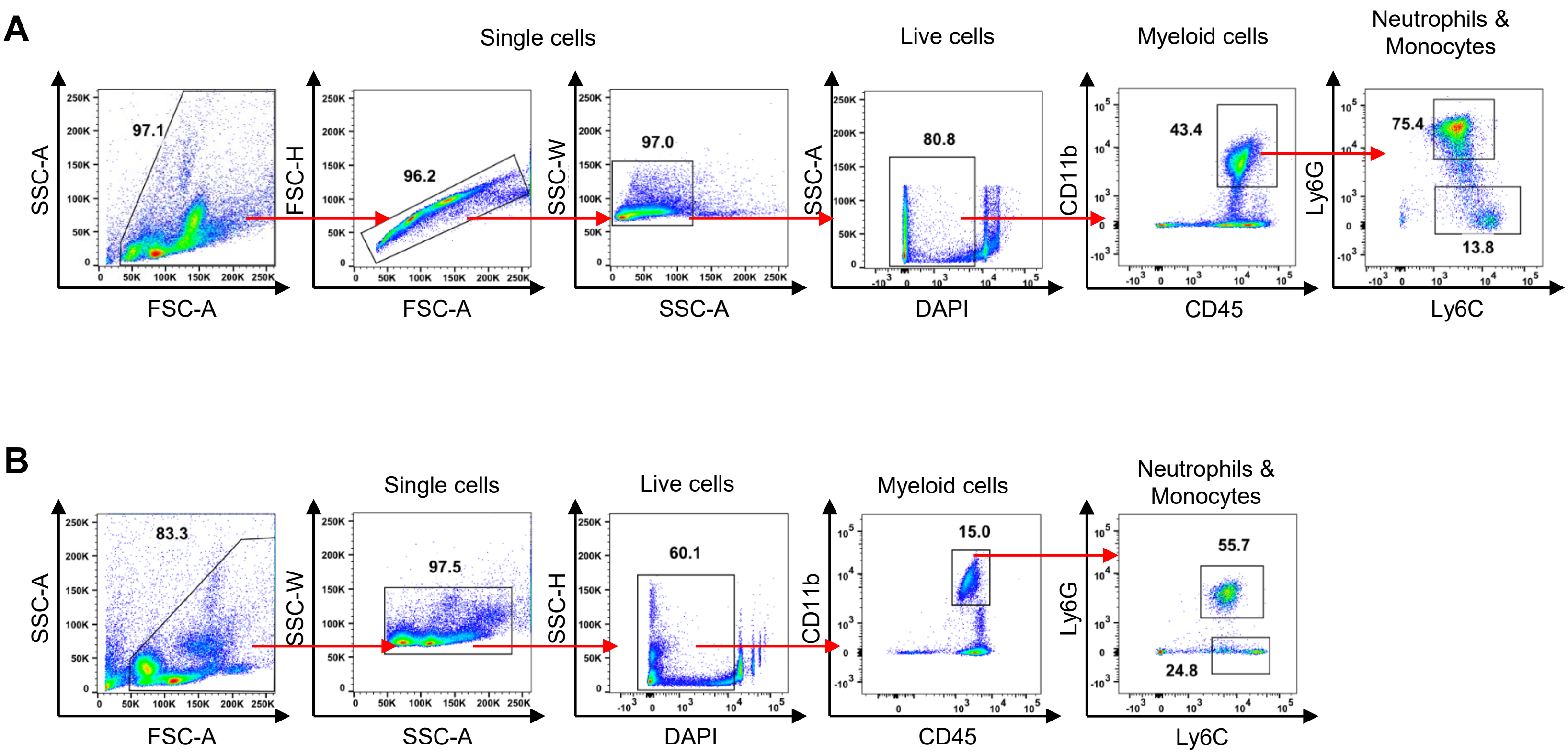

Supplementary Figure S6. Gating strategies of peripheral immune cells in the circulation and bone marrow. (A-B) Full representative gating strategies of flow cytometry analysis of femur bone marrow cells (A) and blood circulatory cells (B) to identify single cells, live cells (DAPI^{-}), myeloid cells, neutrophils, and monocytes, respectively.

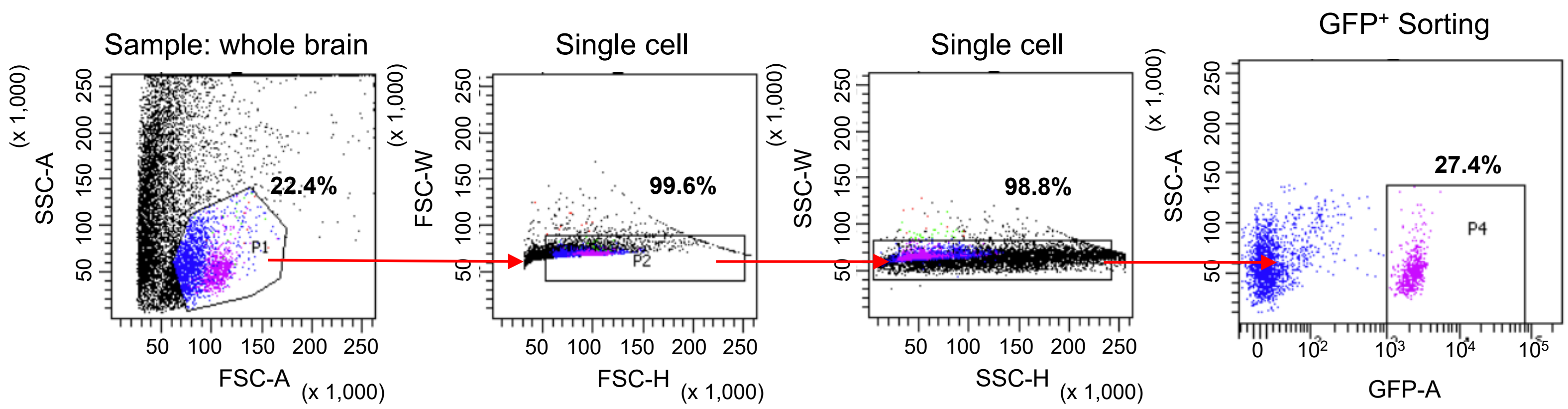

Supplementary Figure S7. Isolation of GFP+ microglia from Tmem119-GFP mouse brain. Representative gating strategies of cell sorting of microglia (GFP+ cells) from brain single cell suspensions of Tmem119-eGFP mice by flow cytometer.

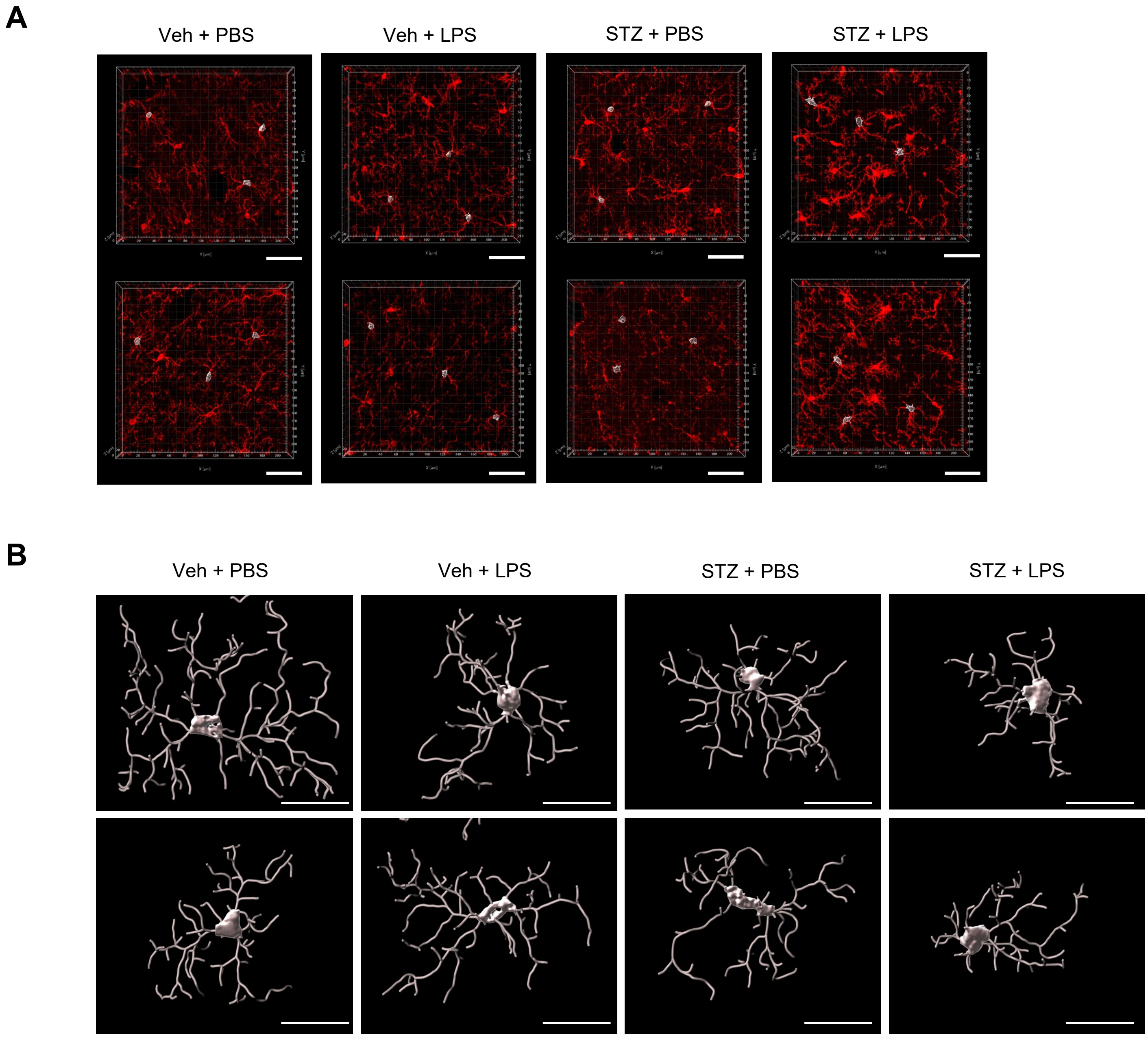

Supplementary Figure S8. Morphological analysis of microglia upon mild inflammatory stimuli in hyperglycemic condition. (A-B) Representative Imaris-processed 3-dimensional confocal images of microglia upon LPS administration in normoglycemic and hyperglycemic mice (LPS 0.5 mg/kg; 6 h). The soma selected for each sample are labeled with white color. Scale bars = 30 μm (A). The filament length and the end points counts of branches are visualized in single-cell resolution. Scale bars = 20 μm (B).

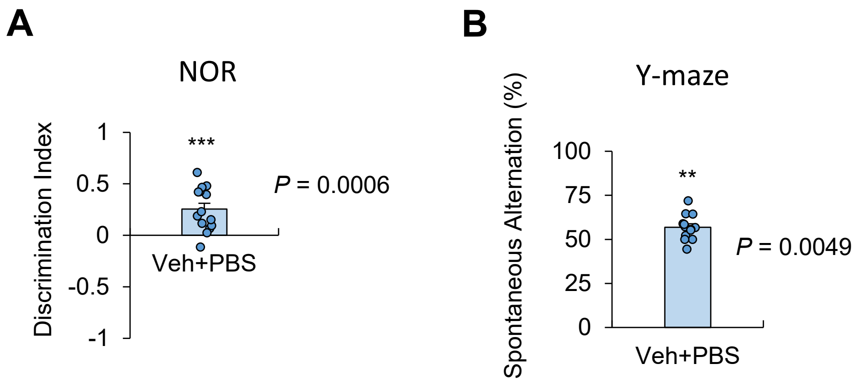

Supplementary Figure S9. Validation of intact cognitive function demonstrated by control mice. (A) One sample *t*-test comparing the discrimination index of the control group (veh-PBS) to a theoretical mean value of 0 in the Novel Object Recognition (NOR) test. (B) One sample *t*-test comparing the spontaneous alternation (%) of the control group to a theoretical mean value of 50% in the Y-maze task. (n = 13 mice) Data are presented as means ± SEM. Asterisks indicate significant differences between the groups as calculated by one sample *t*-test. $^{**}P< 0.01$, $^{***}P<0.001$.
